# Disulfiram treatment suppresses antibody-producing reactions by inhibiting macrophage activation and B cell pyrimidine metabolism

**DOI:** 10.1038/s42003-024-06183-9

**Published:** 2024-04-22

**Authors:** Weili Chen, Etsuko Toda, Kazuhiro Takeuchi, Yurika Sawa, Kyoko Wakamatsu, Naomi Kuwahara, Arimi Ishikawa, Yuri Igarashi, Mika Terasaki, Shinobu Kunugi, Yasuhiro Terasaki, Kazuhiko Yamada, Yuya Terashima, Akira Shimizu

**Affiliations:** 1 Department of Analytic Human Pathology, Nippon Medical School, Tokyo, Japan; 2 Laboratory for Morphological and Biomolecular Imaging, Nippon Medical School, Tokyo, Japan; 3 Division of Molecular Regulation of Inflammatory and Immune Diseases, Research Institute for Biomedical Sciences, Tokyo University of Science, Chiba, Japan; 4 Division of Organ Replacement and Xenotransplantation Surgery, Center for Advanced Biomedical Science and Swine Research, Kagoshima University, Kagoshima, Japan; 5 Division of Pathology, Nippon Medical School Hospital, Tokyo, Japan; 6 Department of Surgery, Johns Hopkins University, Baltimore, MD USA

**Keywords:** Allotransplantation, Metabolic pathways

## Abstract

Antibody responses, involving B cells, CD4 + T cells, and macrophages, are implicated in autoimmune diseases and organ transplant rejection. We have previously shown that inhibiting FROUNT with disulfiram (DSF) suppresses macrophage activation and migration, effectively treating inflammatory diseases. In this study, we investigated the effectiveness of DSF in antibody-producing reactions. Using a heart transplantation mouse model with antibody-mediated rejection, we administered anti-CD8 antibody to exclude cellular rejection. DSF directly inhibited B cell responses in vitro and significantly reduced plasma donor-specific antibodies and graft antibody deposition in vivo, resulting in prolonged survival of the heart graft. DSF also mediated various effects, including decreased macrophage infiltration and increased Foxp3+ regulatory T-cells in the grafts. Additionally, DSF inhibited pyrimidine metabolism-related gene expression induced by B-cell stimulation. These findings demonstrate that DSF modulates antibody production in the immune response complexity by regulating B-cell and macrophage responses.

## Abstract (web summary)

The FDA-approved drug disulfiram can inhibit B cells and infiltration of macrophages in a mouse model of heart transplantation.

## Introduction

Antibody responses play an important role in various autoimmune diseases and organ transplant rejection. Approximately 50% of heart recipients receiving grafts over 7 years developed antibody-mediated rejection (AMR)^1^, which finally resulted in poor long-term graft survival^1,2^. In addition to the presence of donor-specific antibodies, which derived from activated B cell and plasma cells, accumulation of intravascular macrophages, deposition of C4d, have been proposed as pathological diagnostic markers of AMR^3^. Those antigen-specific T cells that primed and activated by antigen-presenting cells, help cognate B cells differentiate into antibody-producing cells^4,5^. Macrophages are activated by antigen-specific helper T cells through the interaction of major histocompatibility complex class II and cognate T cell receptor. They are involved in mediating antibody responses via Fc receptor-mediated cytotoxicity, mediating tissue and cell injuries via inflammatory mediators, and modulating adaptive immune responses via cytokine secretion^6–11^. Several studies have revealed abundant macrophage infiltration in the transplanted graft at an early stage after transplantation^8,12,13^, and it also plays an important role in mediating severe organ damage even graft loss in transplantation rejection^11,14^.

Plasma exchange (PLEX) and immunoadsorption for antibody depletion, intravenous immunoglobulin (IVIG) for immunomodulation, T-cell inhibitory agents, and certain B cell-depletion monoclonal antibody agents have been demonstrated to be beneficial for treating AMR^15^. However, even with these treatments, the activation of B cells and macrophages and the production of antibodies cannot be completely contained. Combination therapy with a single target is complex and prone to uncertainty. Until recently, there has been no approved strategy for multicellular targets of AMR^16^.

FROUNT was originally cloned from a cDNA library of the human monocyte cell line THP-1 to identify CCR2-binding molecules that positively regulate chemotaxis signaling^17^. Later, FROUNT was found to bind to another chemokine receptor, CCR5^18^. As these chemokine receptors mediate monocyte and macrophage migration during inflammation, FROUNT is assumed to be a molecular target for regulating the excess accumulation of macrophages. Our previous study confirmed that disulfiram (DSF), an anti-alcoholism drug, can inhibit the function of FROUNT by blocking the interaction of FROUNT and chemokine receptors CCR2/CCR5 and can inhibit tumor progression and glomerulonephritis^19,20^. Our previous evidence suggests that DSF is an attractive therapeutic target for modulating macrophages to prevent AMR.

In this study, we examined the efficacy of DSF in antibody-producing reactions in vitro and in a mouse heart transplantation model in vivo. For a more accurate understanding of the efficacy of DSF in cardiac allograft rejection focusing on AMR, we depleted CD8 + T cells in mouse heart transplant models, which enables the investigation of mechanisms underlying AMR via CD4 + T cell-dominant immunoreactions.

## Results

### Direct inhibitory effect of DSF on B-cell responses to T-independent and T-dependent stimuli

**Fig. 1:**
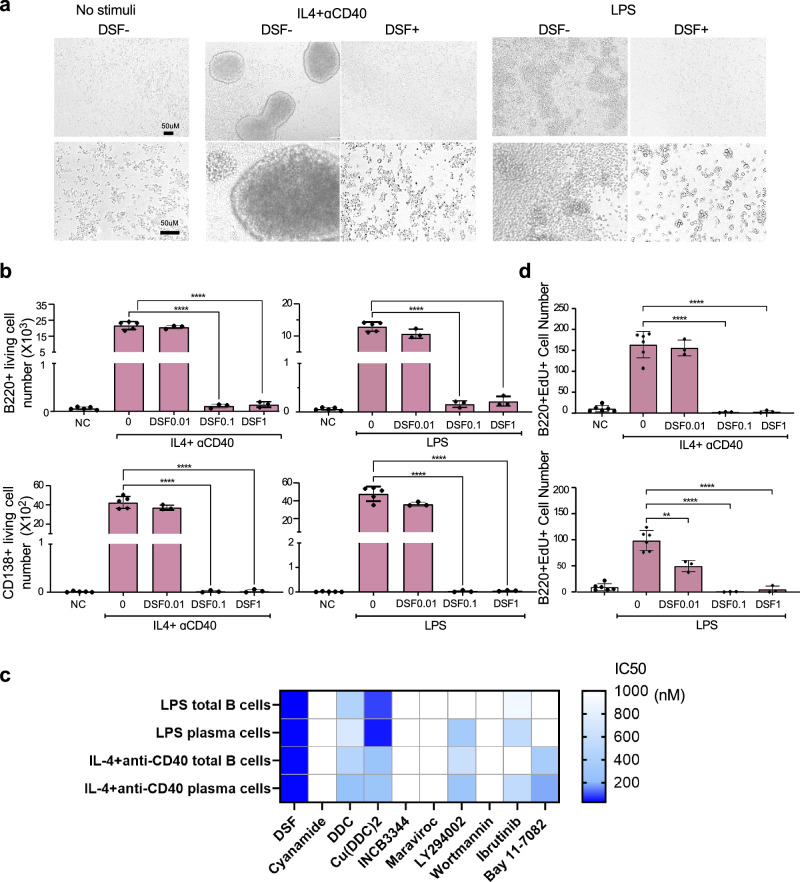
DSF inhibited the B cell response more potently than any other known inhibitors related to B cell function. **a** B-cell cluster formation after stimulation with IL4+anti-CD40 antibody as T-cell-dependent stimuli and LPS as T-cell-independent stimuli. **b** Number of B220 + B cells and CD138+ plasma cells recovered after 4 days of culture in the presence of DSF (NC, unstimulated, *n* = 5; stimulator, *n* = 5; DSF, *n* = 3 biologically independent samples). **c** IC_50_ values (nM) of DSF and DSF-related compounds (cyanamide, DDC and Cu(DDC)_2_), CCR2 or CCR5 inhibitors (INCB3344 and maraviroc, respectively), and known inhibitors for B cell activation (PI3K inhibitor LY294002 and wortmannin, BTK inhibitor Ibrutinib, NF-κB inhibitor Bay11-7082), for the inhibitory effect evaluated by the number of total B cells and plasma cells recovered after 4-day culture with LPS or IL-4+anti-CD40 antibody stimulation. **d** Number of EdU+ B cells recovered after 4 days of culture with IL4+anti-CD40 or LPS in the presence of DSF (NC, *n* = 6; stimulator, *n* = 6; DSF, *n* = 3 independent experiments). Significance was calculated using one-way ANOVA with Tukey’s post-hoc test: **P < 0.01, ****P < 0.0001. Data are shown as the mean ±  SEM.

Before examining the effect of DSF on AMR in the heart transplantation mouse model, we analyzed the direct action of DSF on the B cell response to lipopolysaccharide (LPS) as a T-independent (TI) antigen or IL-4 plus anti-CD40 ligation to induce a T-dependent (TD) response^21^ (Fig. 1a, b). After 4 days of culture, both TI and TD stimulation induced cluster formation of proliferating B cells, and DSF completely blocked B cell cluster formation (Fig. 1a). Flow cytometry analysis of cultured B cells revealed that DSF markedly reduced the number of total B cells as well as the number of CD138+ plasma cells (Fig. 1b).

To explore the molecules involved in the TI and TD responses of B cells and to illustrate the inhibitory mechanism of DSF, we examined the inhibitory effect of known inhibitors on B-cell responses. First, we examined the DSF-related compound, cyanamide, as the more selective aldehyde dehydrogenase (ALDH; EC 1.2.1.3) inhibitor, and diethyldithiocarbamate (DDC) and DDC–copper complex (Cu(DDC)_2_), as metabolism-related products of DSF^19^, in response to stimulation with IL-4+anti-CD40 or LPS (Supplementary Fig. 1). In contrast to the significant inhibition of B cells by DSF at 0.1 μM, no effect was elicited by cyanamide, even at 1 μM. The DSF metabolite DDC inhibited the number of total B cells and plasma cells at 1 μM but not at 0.1 μM, in IL4+anti-CD40 or LPS-stimulated cases. Cu(DDC)_2_ exhibited an inhibitory effect at 0.1 μM, but a weaker effect compared with DSF. Further, based on our previous data, we tested the CCR2 (INCB3344) and CCR5 inhibitors (maraviroc, UK-427857) to explore the involvement of the CCR2 and CCR5 pathways in B cell responses (Supplementary Fig. 2). INCB3344 and maraviroc did not show a significant inhibitory effect on the total number of B cells and CD138+ plasma cells.

We also examined the effects of the inhibition of known pathways involved in B cell activation (Supplementary Fig. 3). The effects of NF-κB/pyroptosis inhibitor Bay 11-7082; BTK inhibitor ibrutinib; and PI3K inhibitors LY294002 and wortmannin on the presence of total B cells (Supplementary Fig. 3a) and CD138+ plasma cells (Supplementary Fig. 3b) after stimulation with IL-4+anti-CD40 or LPS were tested. Of these, ibrutinib reduced the total number of B and plasma cells; the inhibition was lower than that by DSF at 0.1 μM but comparable to that by DSF at 1 μM. Bay 11-7082 and LY294002 inhibited the total number of B and plasma cells only at 1 μM, while wortmannin showed no inhibition at the concentrations tested. Comparing the IC_50_ values, DSF was found to be the most potent among the tested compounds (Fig. 1c).

To elucidate whether a reduced number of B cells or plasma cells is induced by the inhibition of proliferation or the promotion of apoptosis, we evaluated the proliferation and apoptosis markers in B and plasma cells using flow cytometry. DSF markedly reduced the number of proliferative B cells, as detected using EdU staining (Fig. 1d), whereas DSF did not increase the expression of an apoptosis marker, but rather decreased it (Supplementary Fig. 4a). A dilution assay of cell proliferation using flow cytometry with the dilution dye CellTrace also showed that DSF exerted its inhibitory effects by blocking cell division rather than by killing the proliferating cells 48 h after stimulation with either IL-4+anti-CD40 or LPS (Supplementary Fig. 4b). Furthermore, at the earlier time point of 3 h after stimulation, the percentage of apoptotic cells was much lower than that detected after 4 days of culture; moreover, DSF had a minimal effect on the induction of apoptotic cells and even exhibited a protective effect (Supplementary Fig. 4c). This suggests that DSF suppresses the process of proliferation in response to stimulation and has no effect on the induction of spontaneous apoptosis in cells that fail to proliferate.

### DSF alleviated symptoms of AMR, thereby significantly prolonging graft survival in the absence of CD8 + T cells

**Fig. 2:**
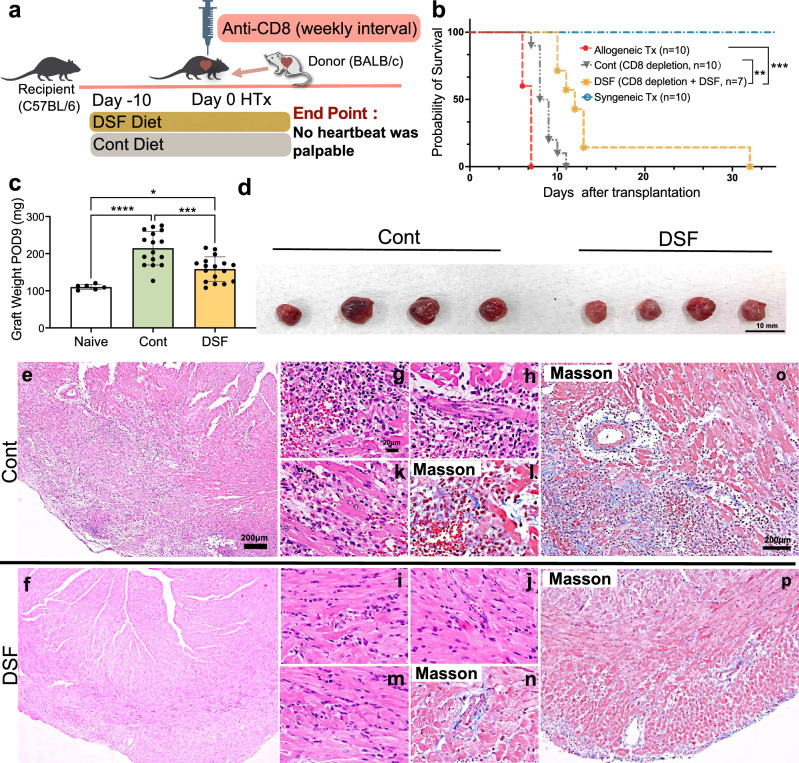
DSF relieving antibodies mediated rejection in murine cardiac transplantation. **a** Experimental design. **b** Survival curves of grafts. Syngeneic heart transplantation (Syngenic Tx, blue line, *n* = 10 biologically independent mice), allogenic heart transplantation (Allogenic Tx, red line, *n* = 10 biologically independent mice), and allogeneic heart transplantation with CD8+ cell depletion combined with the control diet (Cont, gray line, *n* = 10 biologically independent mice) or DSF diet (DSF, yellow line, *n* = 7 biologically independent mice). **c** Graft weight in the naïve group (*n* = 6 biologically independent mice), CD8-depleted group (Cont, *n* = 16 biologically independent mice and CD8-depleted with DSF-treated group (DSF, *n* = 17 biologically independent mice) postoperative day 9 (POD9). **d** Picture of grafts harvested at POD9. **e–p** Hematoxylin-eosin (**e**, **f**, **g**, **h**, **i**, **j**, **k**, **m**) or Masson’s trichrome (**l**, **n**, **o**, **p**) staining of grafts harvested at POD9 from the mice treated with CD8 mAb with control diet (Cont, **e**, **g**, **h**, **k**, **I**, **o**) or DSF-containing diet (DSF, **f**, **i**, **j**, **m**, **n**, **p**). Low-power views (original magnification ×40, **e**, **f**, original magnification ×100 **o**, **p**). 8 high-power views (original magnification ×400, **g**, **h**, **i**, **j**, **k**, **l**, **m**, **n**). Graft survival was compared using log-rank survival statistics (**b**). **P  <  0.01, ***P  <  0.001. Significance was calculated using one-way ANOVA with Tukey’s post-hoc test (**c**): *P < 0.05, ***P < 0.001, ****P   < 0.0001. Data are shown as the mean ± SEM. **a** was generated using Servier Medical Art, licensed under a Creative Commons Attribution 3.0 Unported License.

To test the effect of DSF on AMR and graft survival in a murine cardiac transplant model, we performed heart transplantation using BALB/c (H-2d) heart as the allogeneic grafts or C57BL/6 hearts as the syngeneic grafts in C57BL/6 (H-2b) mice. We depleted CD8 + T cells with anti-CD8 mAb on the day of transplantation to focus more on the DSF-related B-cell- and macrophage-suppressive effects in AMR (Fig. 2a, Supplementary Fig. 5a, b). Grafts in syngeneic transplantation survived for more than 100 days (blue line) (Fig. 2b). Grafts in allogeneic transplantation without any treatment survived for 7 days (red line). When CD8+ cells were depleted (gray line), the allograft survived for 11 days. This suggests that depleting cellular responses mediated by CD8 + T cells can mitigate rejection but is not sufficient to prevent rejection. The administration of DSF through feeding (yellow line) in CD8+ cell-depleted animals significantly prolonged graft survival up to 32 days with an average of 14.4 days, compared with no DSF treatment (gray line) (**P = 0.001). Furthermore, the weight and size of the grafts were lower in DSF-treated mice than in the control mice (Fig. 2c, d). As inflammation-related swelling and edema reflect AMR features, these results indicate that DSF has protective effects against graft rejection, thereby suggesting that DSF may have a suppressive effect on AMR. To further understand the pathological events, we performed histological analysis of the graft tissue on postoperative day 9 (POD9) after transplantation (Fig. 2e–p). Hematoxylin-eosin staining indicated interstitial hemorrhage and interstitial inflammatory cell infiltrate (Fig. 2e, g), severe endothelialitis (Fig. 2h), capillaritis (Fig. 2k), and interstitial edema and hemorrhage (Fig. 2l, o) in the control mouse grafts, which are characteristic features of AMR. These AMR features were relieved in mice treated with DSF (Fig. 2f, i, j, m, n, p).

### DSF alleviates donor-specific antibody levels and complements activation in the graft

**Fig. 3:**
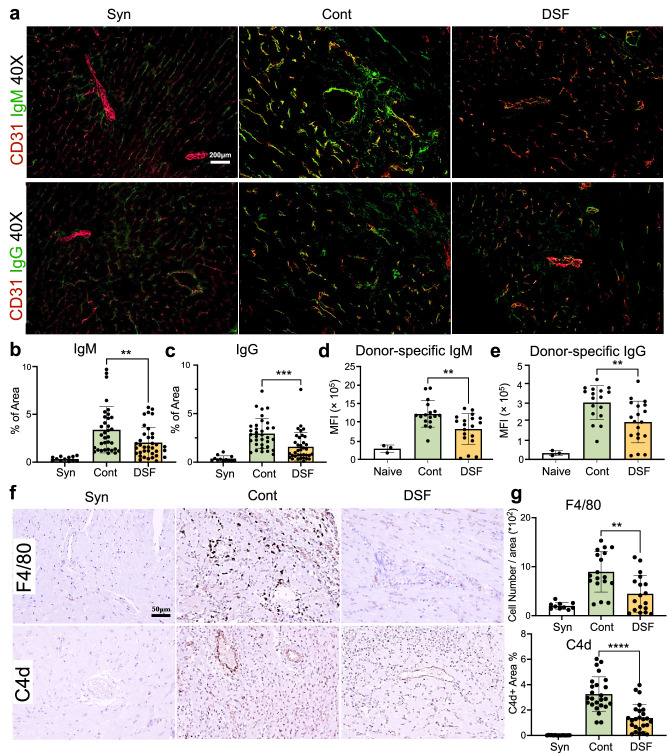
DSF impaired antibody deposition in transplanted grafts and key features of AMR and serum donor-specific antibody level. **a** Immunofluorescence staining image of antibody deposition of IgM (upper line) and IgG (lower line) around the vessel of the grafts (Syn, syngeneic Tx; Cont, allogenic Tx with CD8-depletion and control diet; and DSF, allogenic Tx with CD8-depletion and DSF diet). Quantification of areas positive for IgM (**b**) and IgG (**c**) (Syn; syngeneic, 12 fields in three mouse grafts; Cont, 36 fields in four mouse grafts; DSF, 34 fields in four mouse grafts. Plasma donor-specific antibody IgM (**d**) and IgG (**e**), (Naïve, *n* = 3; Cont, *n* = 17; DSF *n* = 19 biologically independent mice). IHC staining of F4/80 (upper) and complement C4d (lower) of the grafts (**f**). Original magnification ×400. Quantification of the F4/80 positive area (upper) and C4d positive area (lower) in control mice (25 fields in three mouse grafts), DSF mice (25 fields in four mouse grafts) and syngeneic mice (15 fields in three mouse grafts) (**g**). Significance was calculated using a two-tailed, unpaired Student’s *t* test. **P < 0.01, ***P < 0.001, ****P < 0.0001 Data are shown as mean ± SEM.

We then investigated the deposits of IgM (Fig. 3a, upper panel) and IgG (Fig. 3a, lower panel) in the graft. The graft in the control mice showed increased deposition of IgM and IgG compared with the graft of syngeneic transplantation. DSF treatment significantly reduced the positive areas stained with IgM (Fig. 3b, **P = 0.0076) and IgG (Fig. 3c, ***P = 0.0004) in the graft. Plasma donor-specific antibody (DSA) is an established biomarker for predicting AMR^1^. Plasma DSA levels were measured in the recipients on POD 9. DSA levels of both IgM and IgG classes were increased in allogeneic-transplanted mice compared with those in naïve mice without transplantation. We found significantly lower levels of IgM (Fig. 3d, **P = 0.0035) and IgG (Fig. 3e, **P = 0.0046) in the DSF-treated group than in the control group. Furthermore, the key pathological features of AMR, intravascular macrophage accumulation^3,22^ (Fig. 3f, upper), and complement activation product C4d (Fig. 3f, lower), were reduced in the grafts of DSF-treated mice, as evidenced by a significant decrease in the positive area within the grafts (Fig. 3g, **P = 0.0013 and ****P < 0.0001, respectively). Because activated macrophages produce inflammatory cytokines and chemokines to promote immune rejection, we further examined the effect of DSF on cytokine production by activated macrophages using bone marrow-derived macrophages (BMDMs). Macrophages stimulated with LPS secreted cytokines IL-6, MCP-1 (CCL2), TNF-α, IL-12, and IL-1β, all of which were reduced following pre-treatment with DSF in a concentration-dependent manner (Supplementary Fig. 6).

### B cells and plasma cells, in the primary and secondary lymphoid organs of recipient mice, were systemically affected by DSF

**Fig. 4:**
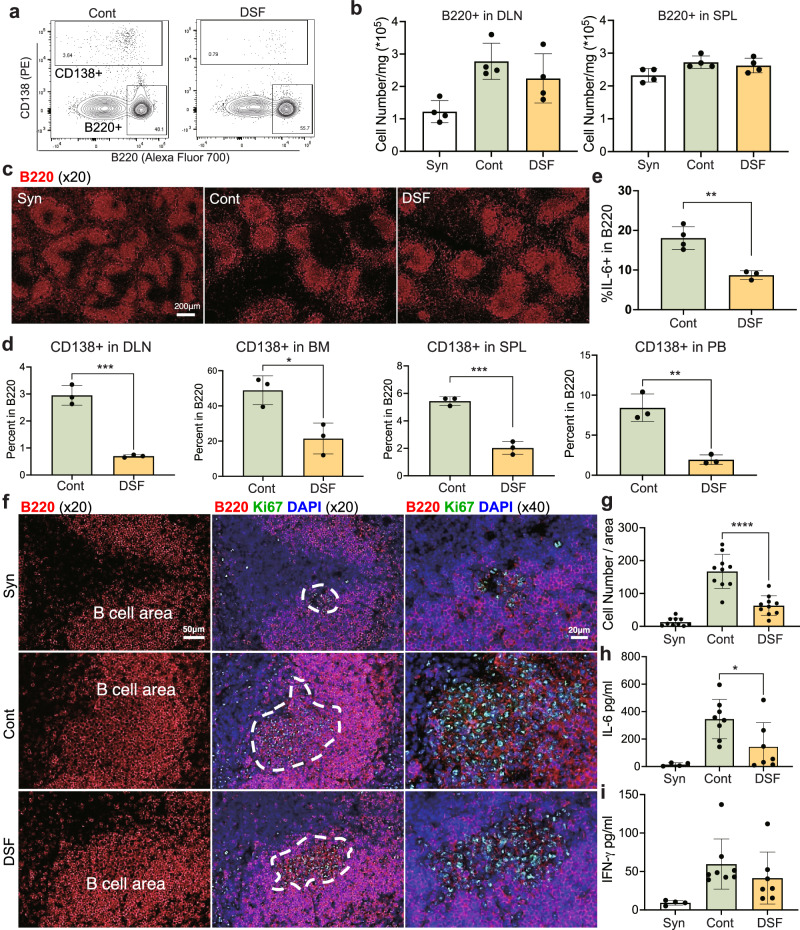
DSF systemically affected B cells and CD138+ plasma cells in vivo at POD9. **a** Flow cytometry plot of B cells (B220+) and CD138+ plasma cells of draining lymph nodes of the control (left) and DSF group (right). **b** The number of B220+ cells in draining lymph node (DLN, *n* = 4 biologically independent mice) and spleen (SPL, *n* = 4 biologically independent mice) (Syn, syngeneic). **c** Immunofluorescent staining of B220 + B cells (red) in the spleen of the recipient. **d** Percentage of CD138+ in B220+ cells in DLN, BM, SPL, and peripheral blood (PB) (*n* = 3 biologically independent mice). **e** Percentage of IL-6+ cells in B220+ cells in the spleen (Cont, *n* = 4; DSF, *n* = 3 biologically independent mice). A representative gating strategy is displayed in Supplementary Fig. 5c. **f** Immunofluorescent staining of Ki67 (green) and B220 (red) of the recipient spleen in syngeneic (Syn, upper), Control group (Cont, middle), and DSF group (DSF, down). **g** Quantification of Ki67 + B220+ cell numbers in the spleen of each recipient (ten fields in three mice). Plasma IL-6 (**h**) and IFN-gamma (**i**) level in recipients (syn, *n* = 4; Cont, *n* = 8; DSF, *n* = 7 biologically independent mice). Significance was calculated using a two-tailed, unpaired Student’s *t* test. *P < 0.05, **P < 0.01, ***P < 0.001, ****P < 0.0001. Data are shown as the mean ± SEM.

We next examined the systemic effects of DSF treatment on the B-cell population in recipient mice (Fig. 4). No significant decline in the number of B220 + B cells was observed in the DSF-treated group in both draining lymph nodes and the spleen (Fig. 4a–c). However, the percentage of CD138+ plasma cells among the B cells (Fig. 4a, d) was significantly reduced in the draining lymph nodes (*P* = 0.0005), bone marrow (*P* = 0.0169), spleen (*P* = 0.0005), and peripheral blood cells (*P* = 0.0035) in the recipients. Furthermore, the expression levels of IL-6 in B cells analyzed by flow cytometry were significantly lower in the DSF group than in the control group (Fig. 4e, *P* = 0.0033). Next, we examined the proliferative capacity of B cells in the spleens of the recipients. Immunohistochemical staining revealed that the DSF-treated group had a reduced proportion of Ki67+ cells in the B cell area (Fig. 4f, g). Further data showed that plasma IL-6, which is mainly derived from activated B cell and macrophages and can promote plasma cell differentiation and suppress the Treg differentiation^23–27^, was significantly reduced in DSF-treated mice (Fig. 4h, *P* = 0.0292). IFN-γ, which is associated with the Th1 response that can inhibit Treg differentiation^28^, was also reduced via the same treatment, although the difference was not significant (Fig. 4i).

### DSF increased the number of CD4+ Foxp3 + CD25+ regulatory T cells in heart graft

**Fig. 5:**
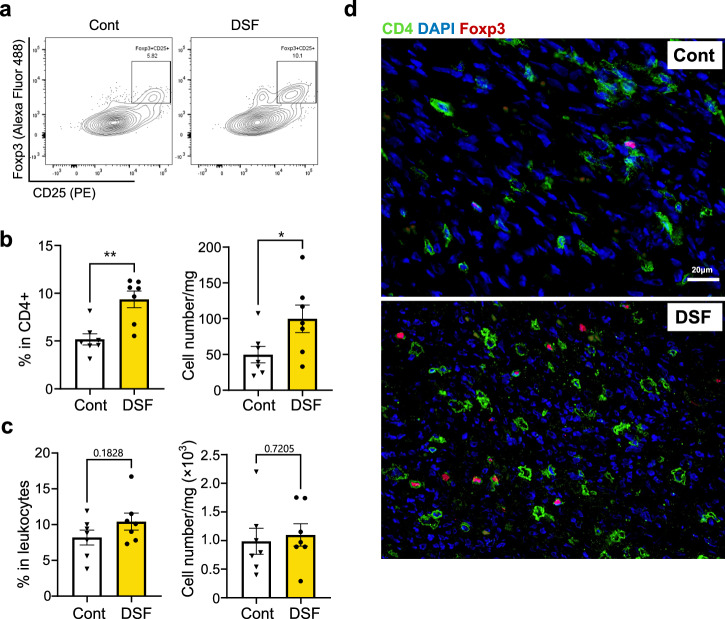
DSF increases the CD4 + CD25+ Foxp3+ regulatory T cell subset in grafts. **a** Flow cytometry plot of CD25+Foxp3+ cells in the CD3 + CD4+ leukocyte gate of the graft-infiltrated cells. **b** Data of FCM profiles of the percentage of CD25+ Foxp3+ cells in CD4+ cells (left) and absolute cell number CD4 + CD25+ Foxp3+ cells (right). *n* = 7 biologically independent mice. **c** Data of FCM profiles of the percentage of CD4 + T cells in leukocytes (left) and absolute cell number CD4 + T cells (right). *n* = 7 biologically independent mice. **d** Immunofluorescent staining of CD4+ (green) and Foxp3+ (red) cells in the graft. Significance was calculated using two-tailed unpaired Student’s *t* test. *P < 0.05, **P < 0.01.

We examined whether DSF alters the subset of CD4 + T cells in CD4 + T cell-dominant heart rejection. Flow cytometry analysis of the heart graft infiltrating cells indicated that both the ratio of CD25+ Foxp3+ regulatory T cells in the CD4+ cell population (**P = 0.0021) and the total number of CD4 + CD25+ Foxp3+ cells (*P = 0.0424) significantly increased in the grafts of DSF-treated mice (Fig. 5a, b), whereas the ratio of CD4 + T cells in leukocytes and the total number of CD4 + T cells did not change (Fig. 5c). Graft staining using immunofluorescence with CD4 (green) and Foxp3 (red) showed more Tregs, which were diffusely distributed in the grafts of DSF-treated mice (Fig. 5d).

### Direct inhibitory effect of DSF on B cell responses was related to the pyrimidine metabolism pathway

**Fig. 6:**
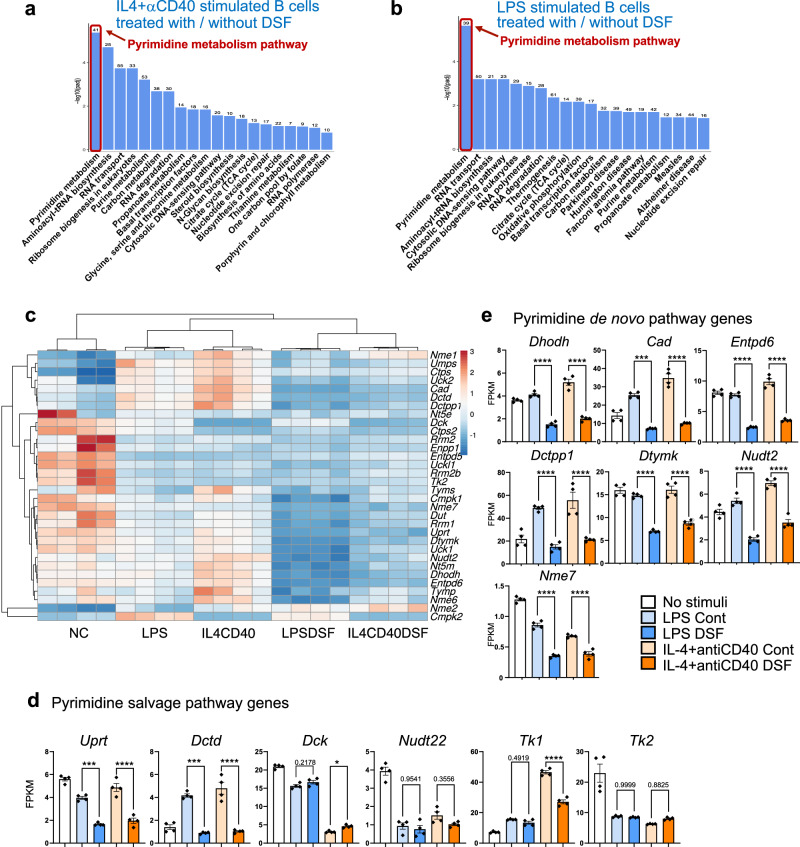
Inhibitory effect of DSF on the B cell response was related to the pyrimidine metabolism pathway. KEGG pathway (down-regulated genes) enrichment analysis of RNA-seq data of B cells 3 h after stimulation by IL4+anti-CD40 antibody (IL4CD40) (**a**) or LPS (**b**), pathways downregulated in DSF-pre-treated B cells compared with those in B cells without DSF. **c** Hierarchical clustering and heat map of mRNA transcript abundance in B cells unstimulated (NC) or stimulated with IL-4 plus anti-CD40 antibody or LPS with DSF (IL4CD40DSF and LPSDSF) or without inhibitor (IL4CD40 and LPS). Gene expression FPKM values obtained from RNA-seq analysis related to salvage pathway (**d**) and de novo pathway (**e**) and in pyrimidine synthesis (*n* = 4 biologically independent samples). Significance was calculated using one-way ANOVA with Tukey’s post-hoc analysis: *P  <  0.05, **P < 0.01, ***P < 0.001, ****P < 0.0001. Data are shown as the mean ± SEM.

To explore the molecules responsible for the inhibitory effect of DSF on B cell activation, we performed a comprehensive gene expression analysis of B cells and DSF-treated B cells following activation with LPS or IL-4+anti-CD40 antibody (3 h post-activation). Pathway analysis of the upregulated gene sets identified in B cells stimulated with either LPS or IL-4+anti-CD40 treatment via comparisons with the expression levels of genes in unstimulated B cells revealed that although the two stimuli upregulated different pathways, ribosome biogenesis pathways were commonly the most significantly upregulated pathways under both stimulation conditions (Supplementary Fig. 7a, b). By contrast, pathway enrichment analysis of the genes with reduced expression levels under DSF treatment showed that the pyrimidine metabolism pathway was significantly downregulated by DSF under both stimulation conditions (Fig. 6a, b). The commonly known target genes in the pyrimidine metabolism pathway^29^ were repressed by DSF (Fig. 6c). We also performed RNA-seq analysis of stimulated B cells in the presence of ibrutinib, a Btk inhibitor (Supplementary Fig. 7c, d), which inhibited B cell responses in a manner comparable to the effect of DSF (with treatment of 1 μM for both in Supplementary Fig. 3). Ibrutinib did not inhibit the expression of genes related to pyrimidine metabolism in B cell activation.

### DSF inhibits the pyrimidine metabolism pathway by de novo synthesis and recycling via the salvage pathway

**Fig. 7:**
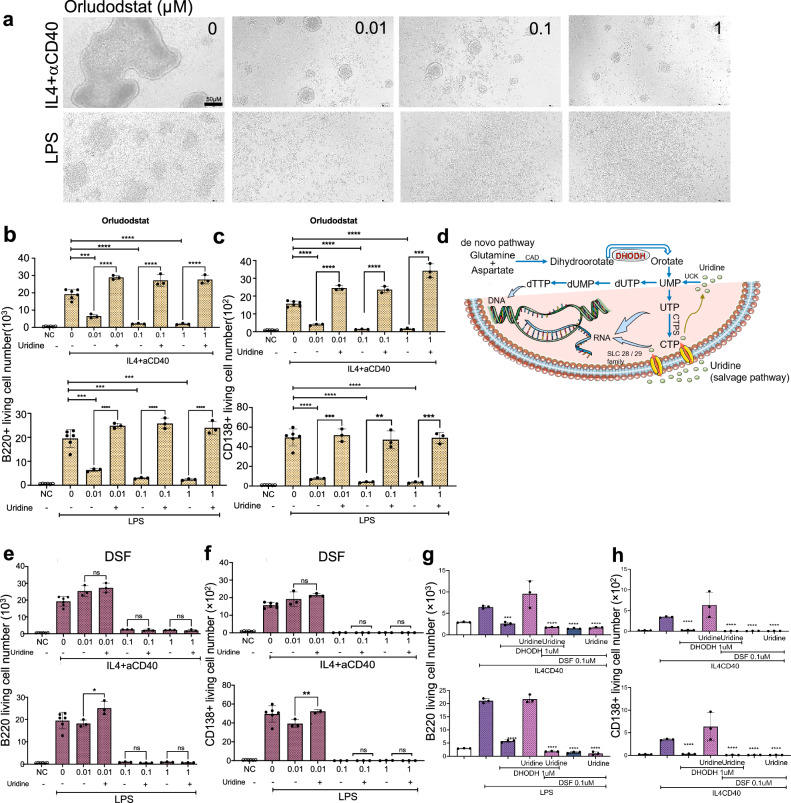
B-cell responses were inhibited by DHODH inhibitor (Orludodstat) comparable to DSF and can be restored by Uridine, but not DSF. **a** B-cell cluster formation after stimulation with IL-4 plus anti-CD40 antibody or LPS with or without pre-treatment with indicated concentrations of Orludodstat. Numbers of B cells (**b**) and CD138+ plasma cells (**c**) recovered after 4-day culture in the presence of indicated concentrations of Orludodstat, stimulated by IL-4 plus anti-CD40 (upper panel) or LPS (lower panel), and effect of uridine added to the B cell culture on the inhibitory effect of Orludodstat (NC, unstimulated, *n* = 6; IL4+anti-CD40, *n* = 6; LPS, *n* = 6; stimulated in the presence of Orludodstat, *n* = 3 biologically independent samples). **d** Simplified schematic of pyrimidine synthesis in de novo synthesis and salvage pathways. Effect of uridine added to the B-cell culture on the inhibitory effect of DSF on B cells (**e**) and CD138+ plasma cells (**f**) recovered after stimulation with IL4+anti-CD40 (upper panel) or LPS (lower panel) (NC, unstimulated, *n* = 6; IL4 + aCD40, *n* = 6; LPS, *n* = 6; stimulated in the presence of DSF, *n* = 3 biologically independent samples). DSF decreased the salvage pathway-driven B cell response induced by inhibition of the de novo pathway by the DHODH inhibitor and addition of uridine in B220+ cells (**g**) or CD138+ plasma cells (**h**) (*n* = 3 biologically independent samples). Significance was calculated using one-way ANOVA with Tukey’s post-hoc test: *P < 0.05, **P < 0.01, ***P < 0.001, ****P < 0.0001. Data are shown as the mean ± SEM. **d** was generated using Servier Medical Art, licensed under a Creative Commons Attribution 3.0 Unported License.

B-cell stimulation upregulates the pyrimidine metabolism pathway; however, the physiological role of this pathway in B-cell activation and differentiation has not yet been elucidated. Therefore, we confirmed the effect of an inhibitor of DHODH, which is the rate-limiting enzyme of the pyrimidine metabolism pathway, on B cell activation. B cells isolated from naïve mice were stimulated with LPS or IL-4 + CD40 ligation in the presence of different concentrations of a potent DHODH inhibitor Orludodstat (also as BAY 2402234). The DHODH inhibitor significantly reduced cluster formation of B cells (Fig. 7a) and the number of total B cells (Fig. 7b). It also reduced CD138+ plasma cells (Fig. 7c) even at a concentration of 0.01 μM. The blockade of de novo pyrimidine synthesis can be reversed by supplying uridine to the salvage pathway (Fig. 7d). Uridine completely restored the reduced number of B and plasma cells in the DHODH inhibitor-treated cases (Fig. 7b, c). In contrast, uridine did not rescue the B cell responses inhibited by DSF (Fig. 7e, f). DSF also decreased the B cell response when the salvage pathway was induced by inhibition of the de novo pathway by treatment with the DHODH inhibitor and addition of uridine (Fig. 7g, h). These results suggest that DSF inhibits both the de novo and salvage pathways in pyrimidine metabolism. Indeed, DSF reduced the expression levels of genes associated with the salvage pathway (Fig. 6d), such as *Uprt* and *Dctd* in both TD and TI responses and *Tk1* in the case of the TD response, in addition to genes related to the de novo pathway (Fig. 6e).

## Discussion

In this study, we first demonstrated that DSF directly inhibited the proliferation of B cells and reduced the frequency of plasma cells in vitro. Building upon these findings, we then applied DSF to a cardiac transplantation model. DSF improved graft survival in a CD4 + T cell-dominant heart rejection model in the transplanted grafts, systemically suppressed plasma DSA levels, and reduced the frequency of plasma cells in vivo. DSF also inhibited macrophage accumulation in the vascular region, alleviated endovascular deposits of IgM and IgG, and increased the number of Tregs in the grafts. Furthermore, DSF inhibited macrophage activation and cytokine secretion that participate in mediating the differentiation of B cells and the production of antibodies. Although these were not confirmed as direct target molecules, our RNA-seq analysis did reveal several candidate molecules with inhibited expression following DSF treatment, which was mainly related to pyrimidine metabolism. Our findings thus indicate the critical role of DSF in regulating AMR progression. As a new therapeutic agent that can suppress AMR by inhibiting B cell activation rather than by eliminating B cells, DSF is expected to find rapid clinical applications, especially as it is already used safely in clinical practice. We also found that the pyrimidine metabolism pathway is involved in the inhibition of B cell proliferation by DSF. Our findings indicate the critical role of DSF in regulating AMR progression.

**Fig. 8:**
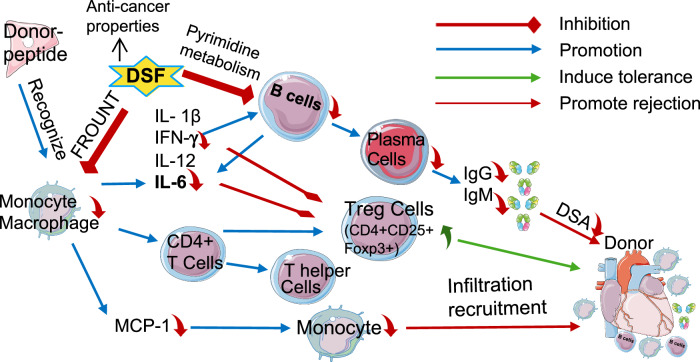
Summary of the effect of DSF on B cells and other rejection-related immune responses. Scheme illustrating the mechanisms of DSF modulating antibody-producing reaction through the regulation of macrophage activation and pyrimidine metabolism in B cells. This figure was generated using Servier Medical Art, licensed under a Creative Commons Attribution 3.0 Unported License.

Survival of oncology and transplant patients has increased significantly with improved treatments. However, cancer patients are at a risk of organ failure, which necessitates transplantation, and transplant recipients receiving long-term immunosuppressive drugs may also develop tumors^30^. Disulfiram with anti-cancer properties^19^ may be a promising therapeutic option that could benefit patients who require complex immune modulation after receiving both transplant immunosuppressants and antitumor therapies (Fig. 8).

In the cardiac transplant model in our study, DSF inhibited macrophage migration and activation, which is consistent with our previous findings in a cancer model^19^ and glomerulonephritis model^20^. This is the first study to show that DSF has a potent effect on B cell and plasma cell inhibition, both in vitro and in vivo (Fig. 1, Supplementary Figs. 1–3). DSF inhibits B cell responses to TI or TD stimulation in vitro more potently than any other inhibitor of molecules involved in B cell activation, suggesting that there may be an undetermined mechanism for the B-cell inhibitory actions of DSF. Although DSF inhibited both TI and TD responses in vitro, we also observed a significant change in the germinal center with reduced proliferation (Fig. 4f), suggesting altered TD responses in the recipient. Apart from the increase in the number of Tregs, the mechanisms mediated by helper T cells, especially follicular helper T cells, should be clarified in the future. Less macrophage infiltration and milder complement deposition were observed in the grafts, as revealed through histological analysis of the grafts (Fig. 3). These findings indicated that DSF can alleviate AMR and suppress endothelial cell activation.

AMR is a major cause of graft loss^2^. Immunosuppressive drugs that are routinely used in clinical practice, such as mycophenolic acid, FK506, and cyclosporine, all have immunomodulatory effects on T cells, including CD8+ cells. This suggests that most cases of AMR occur even when T-cell responses are suppressed by these current drugs. Accordingly, in this study, we depleted CD8 to better focus on the specific effect of DSF on AMR. B cells and plasma cells, as persistent sources of pathogenic alloantibodies and autoantibodies, have been treated as targets for refractory AMR and have gained increasing attention^31^. All treatments, including PLEX, IA, IVIG, immunomodulation, T cells, B cells, or plasma cell depleting agents^15,32,33^, when used individually target the removal of single cells or DSA. The combination is cumbersome and has a limited effect on CD138 + long-lived antibody-producing bone marrow plasma cells^33–36^. Even in recent times, patients with pre-existing HLA antibodies require splenectomy for desensitization before the transplantation^36^. Clinical progress starts with the use of proteasome inhibitors (PIs) to prevent anamnestic AMR with a higher risk^37^. However, PIs are currently limited to preventing anamnestic AMR and are not used for de novo AMR^31^. This evidence highlights the need to upgrade the treatment regimen for AMR.

Previous studies have shown the inhibition of tumor growth by DSF via stress-related signaling pathways such as proteasome inhibition and induction of apoptosis through activation of endoplasmic reticulum stress^38,39^. However, the inhibition in these cases was found to be the most efficient when DSF was present as a copper complex. By contrast, we found that the inhibitory effect on B cells was the most effective with treatment of DSF alone, suggesting that this effect is mediated by a mechanism distinct from that underlying its inhibitory effect on tumor cells. Indeed, RNA-seq and in vitro experiments showed that the pyrimidine metabolism pathway is enhanced upon B cell activation, and DSF inhibits this pathway (Figs. 6, 7). Therapeutic strategies targeting pyrimidine metabolism pathways, which play crucial roles in various cell types, have been investigated in several diseases, including rheumatoid arthritis, multiple sclerosis^40–42^, solid or hematologic tumors^29,43,44^, and organ transplant rejection^45,46^. The DSF-mediated blockade of pyrimidine synthesis interrupts the stimulated B cell proliferation, limiting their expansion and differentiation in antibody-mediated pathogenesis. Most pyrimidine de novo synthesis inhibitors have not shown satisfactory data in clinical trials. This is most likely caused by the salvage pathway which utilizes low-energy depleting nucleosides and helps the inhibited cells escape from the inhibition of the pyrimidine de novo pathway^47^. Recently, superior findings from several studies, which used both inhibitions of de novo pyrimidine and salvage routes for tumor suppression, have received more attention^43,44^. The DHODH inhibitor, BAY 2402234 (Orludodstat), a known pyrimidine metabolism pathway inhibitor, was found to inhibit myeloid malignant cell proliferation. Further evidence showed that this inhibition was rescued by 100 µM of exogenous uridine^48^. By comparing BAY 2402234 with DSF, our study proposes DSF as a novel therapeutic agent that inhibits both the de novo and salvage pathways in pyrimidine metabolism.

Macrophages, together with antibodies and complements, mediate AMR and are involved in acute allograft rejection^1,2,8,10^. Macrophage recruitment in allogeneic mouse transplants typically occurs after myocardial injury and monocyte chemoattractant protein (MCP, also known as CCL2) is involved in the mediation of this process^12^. Our previous findings suggested that DSF inhibits macrophage accumulation by binding to FROUNT to block chemotaxis signaling through CCR2 and CCR5 in macrophages^18,19^. The findings of our current study suggest that DSF can alleviate AMR through two distinct mechanisms: first, pyrimidine metabolic pathway-dependent inhibition of B cells, after which, the resulting antibody reduction alleviates AMR; and second, FROUNT-dependent inhibition of macrophage chemotaxis and cytokine production related to B cell proliferation/activation and differentiation, such as of IL-6 (Supplementary Fig. 6).

IL-6 serves as a marker for the severity of inflammation in various inflammatory diseases and is a target for treatment in numerous clinical studies, including those focused on organ transplantation^23–27,49–52^. Numerous studies have demonstrated that IL-6 promotes severe inflammation and transplantation rejection by promoting the differentiation of effector T cells and plasma cells^26,49,51^, while suppressing the differentiation of regulatory T cells^23,24,26,53^, and IFN-γ is associated with the Th1 response which can inhibit Treg differentiation^28^. Deficiency or neutralization of IL-6 in recipients contributes to allograft tolerance and Treg proliferation^23,25^. IL-6 is secreted by various cells such as mesenchymal cells, endothelial cells, fibroblasts, and tumor cells^51^. Herein, B cells and macrophages were the potential sources of IL-6. The percentage of IL-6+ in B220+ cells was significantly low in DSF-treated mice (Fig. 4e). Bone marrow-derived macrophage-secreted IL-6 was also reduced under DSF treatment in vitro (Supplementary Fig. 6). Macrophages and macrophage-derived factors cross-talk with B cells during normal immune responses^54,55^ as well as in complex transplantation circumstances^56–58^. Our findings suggest that DSF inhibits B cell responses directly and indirectly via inhibiting the production of cytokines related to B cell proliferation/activation and differentiation by macrophages, such as IL-6. The plasma IL-6 levels were also low in the DSF-treated group (Fig. 4h). IL-6 inhibits the induction of Tregs^23^, which are a small subpopulation of immune cells involved in regulating excessive immune activation. The modulation of Tregs is influenced by multiple factors^59,60^. We found that the number of CD4(+) CD25(+) Foxp3(+) Tregs increased in grafts treated with DSF (Fig. 5). Treg cells have been reported to participate in the regulation of AMR^61^ and autoantibody production^62^. A previous study demonstrated that a lower level of serum IL-6 in recipients was associated with an increased percentage of intragraft CD4(+) CD25(+) Foxp3(+) Treg cells and impaired allograft survival^26^.

We consider the lower levels of serum IL-6, which are partially secreted by B cells (Fig. 4e) and by macrophages (Supplementary Fig. 6), in the DSF-treated recipients as one of the major mechanisms contributing to the increased CD4(+) CD25(+) Foxp3(+) Treg cells in the grafts^23^; however, the specific effect of DSF on Treg migration efficiency to the graft should be carefully examined in further studies. Another potential mechanism for the increase in the number of Tregs may involve the direct activation of LCK by DSF reported in the context of antitumor immunity^63^, although further investigations of the transplant rejection mechanism are also required.

The inhibitory activity of the conventional drug DSF against FROUNT to inhibit macrophages and the inhibitory activity of DSF against the pyrimidine metabolism pathway of inhibiting B cells, plasma cells, and DSA provides an effective therapeutic option for AMR. Given that these findings are based on a preclinical model in mice, it is necessary to investigate whether DSF can inhibit the function of long-lived plasma cells or reduce their antibody-producing effects among patients. Considering the limited availability of existing drugs targeting plasma cells, the action of DSF on plasma cells will be further assessed in future studies.

## Methods

### Animals

Female C57BL/6J(H-2b) (7–8-week-old) and BALB/c (H-2d) (5–6-week-old) mice (Japan SLC, Inc. Japan) were used as heart transplant recipients and donors, respectively. The study complies with the ARRIVE guidelines for mouse experiments. All animal experiments were approved by the Animal Experiments Ethical Review Committee of Nippon Medical School (Tokyo, Japan, No. 2019-048). We have complied with all relevant ethical regulations for animal use.

### Heterotopic heart transplantation

We performed heterotopic heart transplantation using C57BL/6 (syngeneic donors) or BALB/c (allogeneic donors) mice to C57BL/6 mice. For testing heart graft survival in a mouse heart transplant model, we divided mice into the following four groups with a randomization sequence: syngeneic transplantation (*n* = 10), allogeneic transplantation (*n* = 10), anti-CD8 monoclonal antibody with control diet (*n* = 10, control diet consistent with DSF-containing diet except for DSF), and anti-CD8 mAb with DSF-containing diet (1.6 mg DSF/1 g) (*n* = 7). We started the control diet or DSF diet feeding from 10 days before the surgery day until the endpoint (graft rejection or scheduled euthanasia). Anti-CD8 mAb (clone 2.43, BioXCell, NH, USA) was injected intraperitoneally (Ip) on days 0 and 7 (0.2 mg/animal). The grafts were assessed daily through palpation to evaluate graft survival. Other mice were euthanized by Isoflurane inhalation, followed by cervical dislocation on POD9 for histological examination and in vivo experiments.

### Surgical procedures of heart transplantation

Hearts from BALB/c mice were transplanted into the neck vessels of all recipients using a non-suture cuff technique^64^. Vascularized heterotopic transplantation of the cardiac allografts was performed using microsurgical techniques. Briefly, 0.2 mL of cold heparin (Mochida Pharmaceutical Co. Ltd., Tokyo, Japan; Heparin Sodium Injection 5000 units/5 mL) was infused into the peritoneal cavity of donor mice. Hearts were harvested following dissection of the ascending aorta and pulmonary artery. The harvested donor hearts were stored at 4 °C and immersed in 0.9% saline with heparin (Mochida Pharmaceutical Co. Ltd.; 5000 units/5 mL) until transplantation. After a supraclavicular incision of the recipient mouse, the internal carotid artery and vena jugularis externa cava were clamped. The ascending aorta and pulmonary artery of the donor’s heart were attached to the cannulated internal carotid artery and vena jugularis externa of the recipient mouse using a non-suture cuff technique^64^. Beating of the transplanted heart was observed upon removal of the cross-clamp, and the incision was closed. The survival of the cardiac allografts was assessed through daily heartbeat palpation. Rejection was defined as the complete cessation of cardiac contractility, as determined via direct visualization and confirmed via histology.

### Histological analysis

On POD9 following heart transplantation, at least three animals in each group were humanely sacrificed under anesthesia. The middle part of the heart tissue was then taken for histological evaluation. The grafts for histological analysis were fixed in 10% buffered formalin and embedded in paraffin. Transventricular tissue sections (2.0-μm thick) were stained with hematoxylin and eosin or Masson trichrome for histopathological examination. For immunohistochemistry, the following primary antibodies were used: C4d (HP1088, Hycult Biotech, USA) for detecting complement, F4/80 (70076, clone D2S9R, dilution 1:100, Cell Signaling Technology, Beverly, MA, USA) for detecting graft-migrated macrophages, and CD8 (ab209775, clone EPR20305, dilution 1:100, Abcam, Cambridge, UK) to confirm successful depletion of CD8 T cells.

### Immunofluorescence staining

The mice were anesthetized, and the grafts were isolated. The base of the heart grafts was embedded in optimal cutting temperature compound (Sakura Finetechnical) and frozen in liquid nitrogen. Fresh-frozen tissue sections (5-μm thick) were prepared and fixed with acetone. After immersion in phosphate-buffered saline (PBS), the sections were stained with antibodies against Ki67 (718071, clone sp6, ready-to-use, Nichirei Biosciences, Japan), B220 (103202, clone RA3-6B2, dilution 1:100, BioLegend, Japan), CD31 (102432, clone 390, dilution 1:100, BioLegend), Alexa Fluor 480-conjugated IgM (ab150121, dilution 1:200, Abcam), and Alexa Fluor 480-conjugated IgG (a-21202, dilution 1:200, Thermo Fisher Scientific, Waltham, MA, USA). Paraffin sections were stained with anti-mouse CD4 antibody (ab237722, clone CAL4, dilution 1:100, Abcam) and Foxp3 (14-5773-82, clone FJK-16s, dilution 1:100, eBioscience, San Diego, CA, USA), followed by staining with Alexa Fluor 488-conjugated anti-rabbit IgG antibody (A21202, dilution 1:200, Thermo Fisher Scientific) and Alexa Fluor 594-conjugated anti-rat IgG (A21209, dilution 1:200, Thermo Fisher Scientific). Images of the transplanted mice stained with antibodies were acquired using a fluorescence microscope (Olympus BX53, Tokyo, Japan).

### Quantification of histopathologic findings

Graft IgM or IgG deposition in 11 mice (12 vessel fields in three mice in the syngeneic group, 34 fields in four mice in the control, and 36 fields in four mice in the DSF group) was counted. Twelve vessel fields were counted in syngeneic grafts, 34 in control grafts, and 36 in DSF grafts. The graft from each mouse was evaluated and expressed as a percentage using WinROOF software).

The number of B220 + Ki67+ in 10 B-cell area fields of the recipient spleen in six mice (two mice from each group) was counted. Cell number was evaluated and expressed using WinROOF software.

The number of F4/80+ macrophages was counted in 10 vessel fields of three syngeneic grafts, 18 of three control grafts, and 18 of four DSF grafts. The data were evaluated as cell numbers and quantified using QuPath software (version 0.3.2).

### Flow cytometry

Spleens, draining lymph nodes and bone marrow from femurs were harvested and mashed through a 70-μM cell strainer, and red blood cells were lysed in ammonium-chloride-potassium (ACK) lysis buffer. After harvest, the middle part (one-third) of the heart was cut into approximately 2 mm fragments and placed in RPMI-1640 medium containing 1 mg/mL collagenase D (Sigma-Aldrich, St. Louis, MO, USA), 10 mM HEPES (Thermo Fisher Scientific), and 0.1 mg/mL DNase I (Thermo Fisher Scientific) and incubated at 37 °C for 60 min to allow for tissue digestion. Peripheral blood cells were treated with ACK lysis buffer, and debris was removed by passing the cells through a 70-µm cell strainer. The cells were resuspended in 2% fetal bovine serum (FBS) in PBS for cell counting and flow cytometry staining. Cells were stained with Zombie-aqua (BioLegend) to discriminate dead cells, incubated with anti-CD16/CD32 antibody (Bio X Cell) to block Fc receptors, and labeled using fluorescence-conjugated anti-mouse antibodies: CD3ε -PE/Dazzle 594 (clone 145-2C11, dilution 1:200, Cat 100347), CD4-Pacific blue (clone RM4-4, dilution 1:200, Cat 116008), B220-AlexaFluor700 (clone RA3-6B2, dilution 1:200, Cat 103232), CD138-PE (clone 281-2, dilution 1:200 Cat 142504), and CD8-APC/Fire™ 750 (clone 53-6.7, dilution 1:200 Cat 100766). Data were acquired using a flow cytometer (LSR Fortessa; BD Biosciences, San Jose, CA, USA).

### Intracellular cell staining

Splenocytes were stimulated using Cell Activation Cocktail (with Brefeldin A) (BioLegend) at 37 °C and 5% CO_2_ for 5 h. Cells were then collected, and dead cells were stained with Zombie Aqua staining (BioLegend). The Intracellular Fixation & Permeabilization Buffer Set (eBioscience) was used for intracellular cytokine staining. Cells were fixed for 20 min at 37 °C. Permeabilized cells were then stained with IL-6-APC (clone RM4-4, dilution 1:200, BioLegend, Cat 116008). For Foxp3 staining to evaluate regulatory T cells, True-Nuclear Transcription Factor Buffer Set (BioLegend) was used. Briefly, splenocytes or draining lymph node lymphocytes were collected and fixed for 45 min, washed with permeabilization buffer, and stained with Alexa Fluor 488-conjugated anti-Foxp3 and isotype control antibodies (eBioscience), followed by surface staining with CD4-Pacific blue (clone RM4-4, dilution 1:200, Cat 116008), CD25-PE (clone 7D4, dilution 1:200, BD biosciences, Cat 558642), CD3ε -PE/Dazzle 594 (clone 145-2C11, dilution 1:100, Cat 100347) and CD45-APC (clone 30-F11, dilution 1:400, BioLegend, Cat 103112) at 4 °C for 30 min. Data were acquired using FACS LSR Fortessa (Becton, Dickinson and Inc.) and analyzed using the FlowJo Software 10.6.2 (BD Biosciences).

### Cytokine measurements

Plasma collected using heparin as an anticoagulant was used for analysis of plasma cell cytokines. Plasma was collected after centrifuging the blood sample for 5 min at 800 × *g* and stored at −20 °C. Plasma and BMDM-secreted cytokines were measured using a LEGENDplex Bead-assisted multiplex cytokine kit (BioLegend). The data were acquired on a flow cytometer (Cytoflex; Beckman Coulter Inc., Brea CA, USA) and analyzed with LEGENDplex Data Analysis Software (BioLegend).

### Plasma donor-specific alloantibody detection

Splenocytes from BALB/c mice were prepared as described above. After incubation with Zombie-Aqua (BioLegend) for dead cell staining and anti-CD16/CD32 antibody (Bio X Cell) to block Fc receptors, the cells were incubated with 20 µL recipient plasma for 30 min. After washing, the splenocytes were incubated with Alexa Fluor 488-conjugated anti-mouse IgG (A-21202, Thermo Fisher, 1:150) or Alexa Fluor 488-conjugated anti-mouse IgM (ab150121, Abcam, 1:150) immunofluorescent antibody for another 30 min. Data were acquired using a flow cytometer (CytoFlex; Beckman Coulter, Inc.).

### BMDM culture experiments

BMDMs were obtained by culturing bone marrow cells obtained from C57BL/6 mice for 7 days in 10% FBS-containing RPMI 1640 medium supplemented with 10% L929 cell culture supernatant. BMDMs were stimulated with 1 μg/mL LPS (Escherichia coli O111: B4; Sigma-Aldrich). After 24 h, the culture supernatants were harvested and subjected to cytokine measurement.

### Cultures for B cell proliferation

B cells were isolated by negative selection using the MACS Pan B cell isolation kit II (Miltenyi Biotech, USA) from C57BL/6 splenocytes and cultured in 96-well flat-bottom plates (Corning, Corning, NY, USA) at 1 × 10^5^ cells/well in 200 µL/well in a humidified atmosphere of 5% CO_2_ at 37 °C. B cells were stimulated with 10 μg/mL LPS (Escherichia coli O127: B8; Sigma-Aldrich)^21^ or stimuli including 100 U/mL IL-4 (Peprotech, Rocky Hill, NJ, USA) and 1 μg/mL anti-CD40 (clone 1C10; BioLegend)^65^. The following compound were added to the B cell culture: disulfiram (NOCBIN, Mitsubishi Tanabe Pharm Corp., Tokyo, Japan), cyanamide (187364, Sigma-Aldrich), diethyldithiocarbamate (DDC, 228680, Sigma-Aldrich), DDC–copper complex (Cu(DDC)2, D0487, Tokyo Chemical Industry Co., Ltd., Tokyo, Japan), INCB3344 (S8220, Selleck, Houston, TX, USA), Maraviroc (UK-427857, S2003, Selleck), Bay 11-7082 (S2913, Selleck), ibrutinib (PCI-32765, HY-10997, MedChemExpress, Monmouth Junction, NJ, USA), LY294002 (B-0294, Echelon Biosciences Inc., Salt Lake City, UT, USA) and wortmannin (A11161, Adooq Bioscience, Irvine, CA, USA) and orludodstat (BAY 2402234, S8847, Selleck) and uridine (U3003, Sigma-Aldrich).

After 96 h, cell cluster images were captured using a microscope (OLYMPUS IX71). The cells were harvested and labeled using conjugated antibodies, and the corresponding isotype antibodies were used for multi-color flow cytometric staining and analyses (CytoFlex; Beckman Coulter Inc.). The number of live cell events per one-minute recording at the medium flow rate in the cytometer under the same suspension volume was used as the cell number for comparison.

### Staining of apoptotic and proliferating cells

Apoptotic cells were stained using ApoTracker Green (BioLegend). Proliferating cells were analyzed using the Click-iT Plus EdU Alexa Fluor 647 Flow Cytometry Assay kit (Thermo Fisher Scientific) according to the manufacturer’s instructions. For dilution assay to detect cell division, isolated B cells were stained with CellTrace Far-red staining solution (Thermo Fisher Scientific) according to the manufacturer’s instructions and cultured as described above. After 48 h, cells were harvested and stained with ApoTracker Green. Data were obtained using CytoFlex (Beckman Coulter) and analyzed using the FlowJo software 10.6.2 (BD Biosciences).

### RNA-seq sample preparation

B cells were isolated by negative selection using the MACS Pan B cell isolation kit II (Miltenyi Biotech) and seeded at 4 × 10^6^ cells/well in 48-well plates. We tested five types of treatment groups: naïve B cells without stimulation and without inhibitor (NS_DMSO), stimulated with LPS without inhibitor (LPS_DMSO), stimulated with IL-4 + anti-CD40 without inhibitor (IL4CD40_DMSO), stimulated with LPS and treated with 1 μM DSF (LPS_DSF), and stimulated with IL-4 + anti-CD40 and treated with 1 μM DSF (IL4CD40_DSF). RNA was extracted from the cell samples using the RNeasy Mini kit (Qiagen) and frozen at −80 °C for next-generation RNA sequencing (Novogene, Beijing, China). Messenger RNA was purified from total RNA using poly-T oligo-attached magnetic beads. The cDNA library was generated and checked with Qubit and real-time PCR for quantification and a bioanalyzer for size distribution detection. Quantified libraries were pooled and sequenced on Illumina NovaSeq 6000.

### Quantification of gene expression level

Sequence reads were trimmed for adapter sequence/low-quality sequence using Fastp (remove reads containing *N* > 10% ; remove reads when low-quality nucleotides (Base Quality less than 5) constitute more than 50% of the read). Trimmed sequence reads were mapped to mm10 using HISAT2 v2.0.5 (parameter: Default). featureCounts v1.5.0-p3 was used to count the reads numbers mapped to each gene. Then FPKM of each gene was calculated based on the length of the gene and reads count mapped to this gene. Reference genome and gene model annotation files were downloaded from the genome website directly.

### Enrichment analysis of differentially expressed genes

Kyoto Encyclopedia of Genes and Genomes (KEGG) is a database resource for understanding high-level functions and utilities of biological systems, such as cells, organisms, and ecosystems, from molecular-level information, especially large-scale molecular datasets generated by genome sequencing and other high-throughput experimental technologies (http://www.genome.jp/kegg/). We used the cluster profile R package to test the statistical enrichment of differentially expressed genes in KEGG pathways. Genes showing up- and downregulation were clustered and visualized as heatmaps using ClustVis^66^ (https://biit.cs.ut.ee/clustvis/).

### Statistics and reproducibility

Statistical analyses were performed using the GraphPad Prism software ver.9. Immunology assay results were compared using the Student’s *t* test and one-way ANOVA with Tukey’s post-hoc test. Graft survival was compared using log-rank survival statistics. The experiments were conducted independently at least three times. *P* < 0.05 was considered statistically significant (*P < 0.05, **P < 0.01, ***P < 0.001, ****P < 0.0001). Data are shown as the mean ± SEM.

### Reporting summary

Further information on research design is available in the Nature Portfolio Reporting Summary linked to this article.

### Supplementary information

Supplementary information

Description of Additional Supplementary Materials

Supplementary Data 1

Reporting summary

## Data Availability

RNA sequencing data have been deposited in the NCBI GEO and are accessible through the accession number GSE261712. The source data underlying the graphs in the article and supplementary information file can be found in Supplementary Data. Further information is available from the corresponding author upon reasonable request.
