# Supplementary material for: Disulfiram treatment suppresses antibody-producing reactions by inhibiting macrophage activation and B cell pyrimidine metabolism
Source: Commun Biol. 2024 Apr 22;7:488. doi: 10.1038/s42003-024-06183-9 (PMC11035657; doi:10.1038/s42003-024-06183-9)
Supplement: Supplementary file 1 — Supplementary information [file 42003_2024_6183_MOESM1_ESM.pdf]

Supplementary Information

Disulfiram treatment suppresses antibody-producing reactions by inhibiting macrophage activation and B cell pyrimidine metabolism

Weili Chen, Etsuko Toda, Kazuhiro Takeuchi, Yurika Sawa, Kyoko Wakamatsu, Naomi Kuwahara, Arimi Ishikawa, Yuri Igarashi, Mika Terasaki, Shinobu Kunugi, Yasuhiro Terasaki, Kazuhiko Yamada, Yuya Terashima, Akira Shimizu

Supplementary Figures 1-7

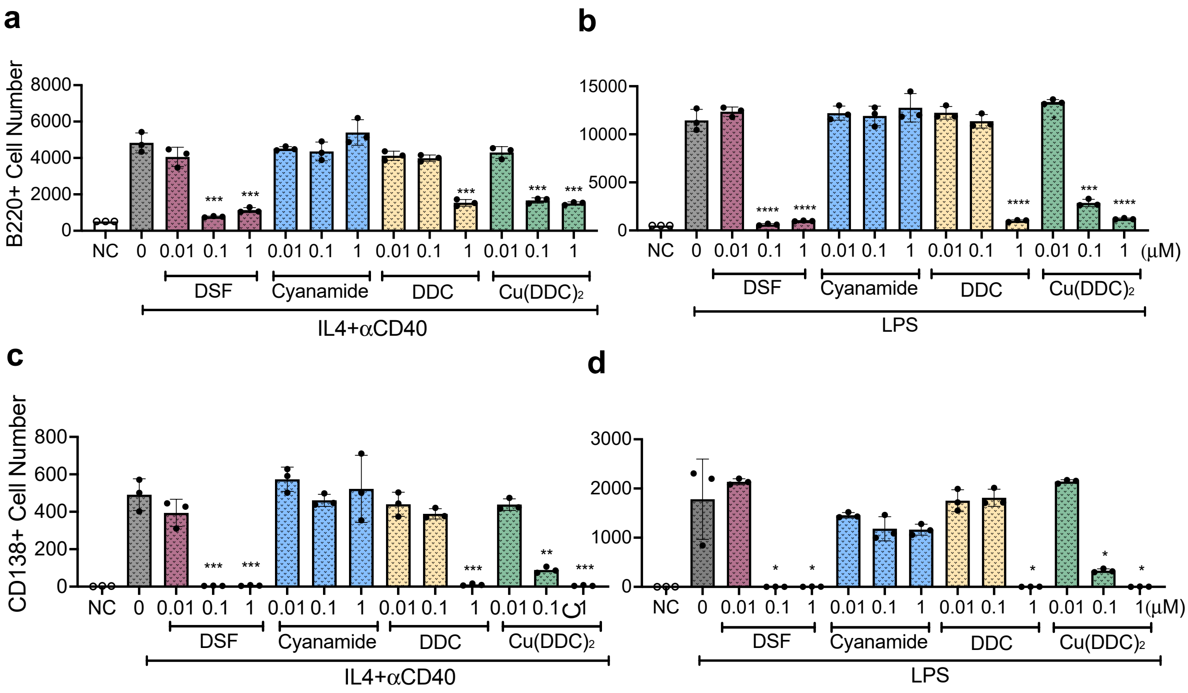

Supplementary Figure 1. B cell responses were potently inhibited by DSF rather than its related compound.

DSF, cyanamide, DDC, and Cu $(DDC)_2$-pretreated B cells stimulated by IL4+anti-CD40 antibody (**a, c**) or LPS (**b, d**). The number of B220+ cells (**a, b**) and CD138+ cells (**c, d**) was reduced by DSF at low concentration (0.1 μM), and to a lesser extent by Cu (DDC)2 at 0.1 μM, whereas the cells were inhibited by DDC at 1 μM. Cyanamide, an aldehyde dehydrogenase inhibitor, cannot B220+ and CD138+ cell numbers. (NC, unstimulated, n=3; Stimulator, n=3; DSF, n=3; Cyanamide, n=3; DDC, n=3; $Cu(DDC)_2$, n=3), Significance was calculated using one-way ANOVA: $^*P < 0.05$, $^{**}P < 0.01$, $^{***}P < 0.001$, $^{****}P < 0.0001$. Data are shown as the mean ± SEM.

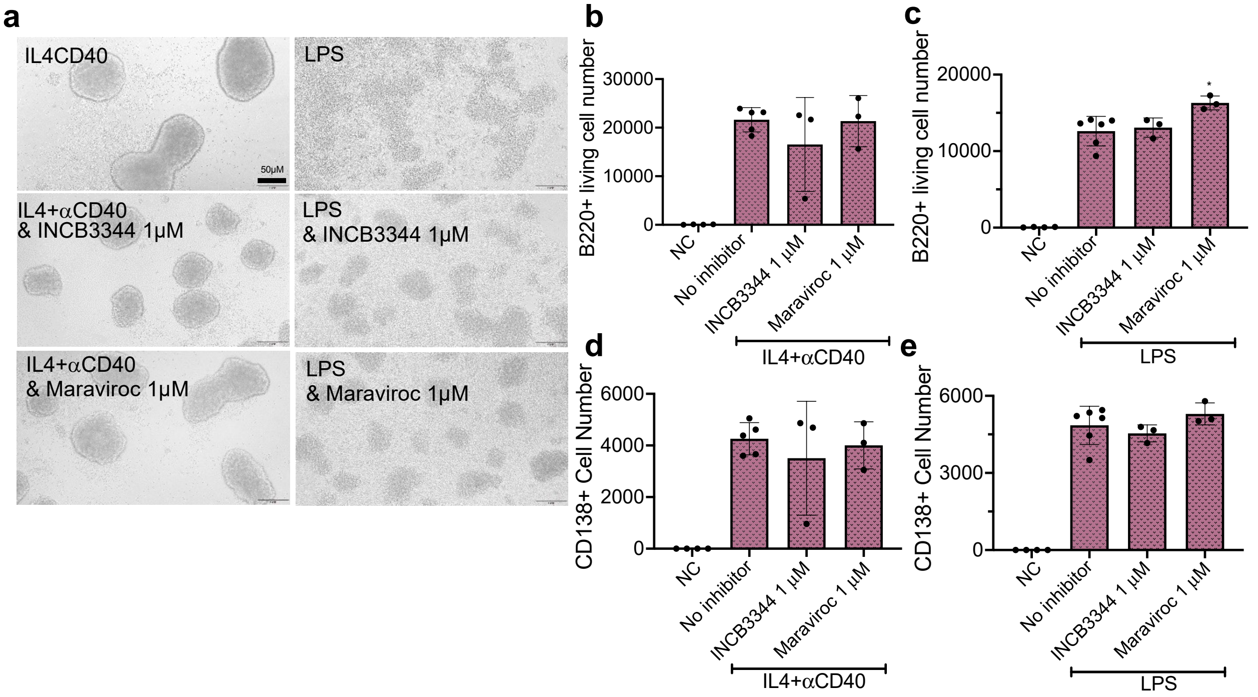

Supplementary Figure 2. CCR2 or CCR5 inhibition did not inhibit B cell responses. a B cell cluster formation after stimulation with IL4+anti-CD40 antibody (left row) and LPS (right row). **b-e** Number of B cells and CD138+ plasma cells recovered after 4-day culture in the presence of the CCR2 inhibitor INCB3344 and CCR5 inhibitor Maraviroc stimulated by IL-4 plus anti-CD40 (**b, d**) or LPS (**c, e**) (NC, no stimulation, n=4; , stimulator alone, n=5; stimulator with INCB3344, n=3; stimulator with Maraviroc, n=3). Significance was calculated using one-way ANOVA but no significant difference was identified. Data are shown as mean ± SEM.

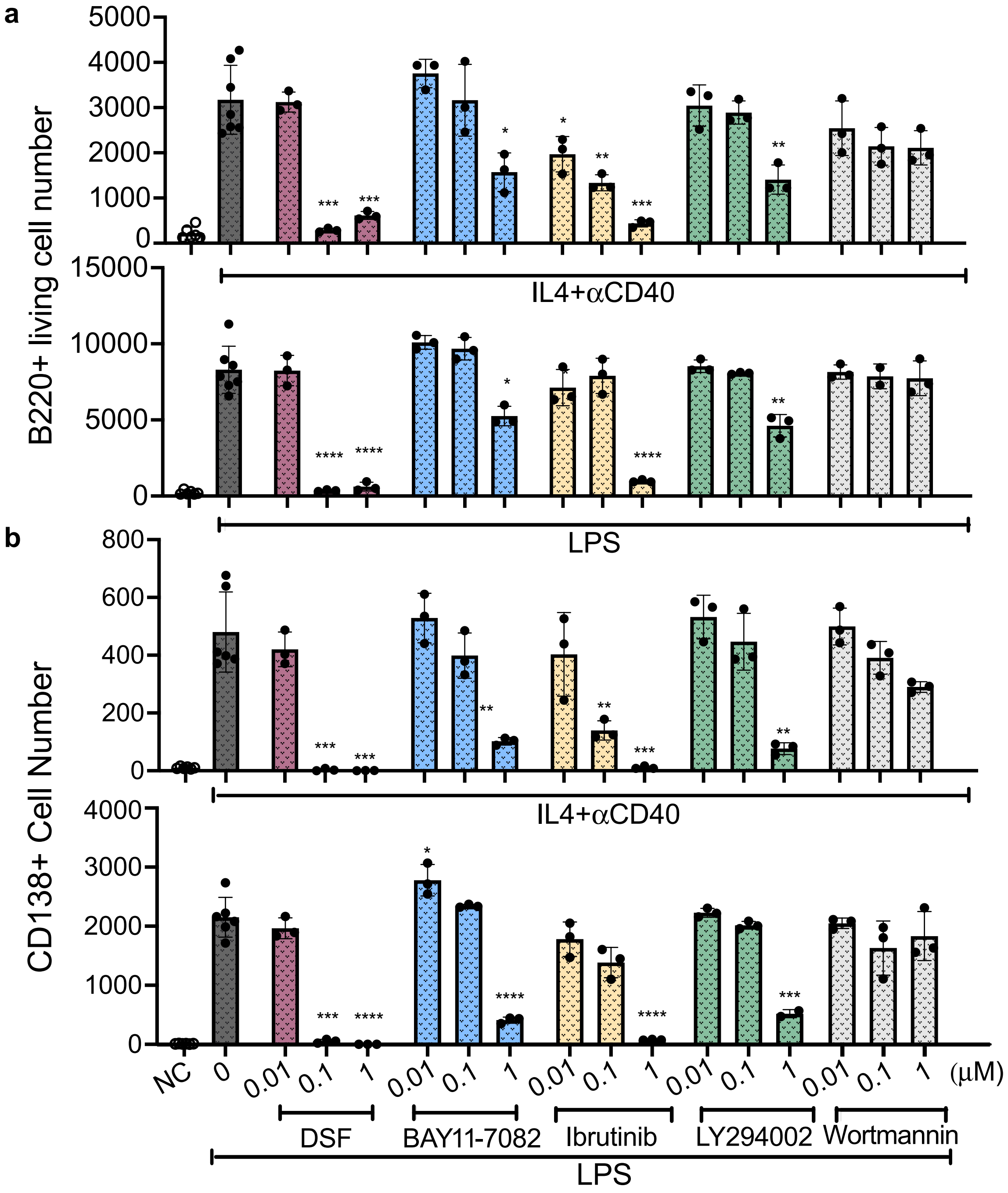
a
b
B220+ living cell number
CD138+ Cell Number
IL4+αCD40
LPS
NC
0
0.01
0.1
1
(μM)
DSF
BAY11-7082
Ibrutinib
LY294002
Wortmannin

Supplementary Figure 3. Inhibitory effect of DSF on B cell response to TD and TI stimulation was stronger than NF-kB inhibitor (Bay11-7082), BTK inhibitor (Ibrutinib), PI3K inhibitor (LY294002, Wortmannin). a, b B-cells isolated from naïve mouse spleens without any inhibitor and pretreated with DSF, Bay11-7082, Ibrutinib, LY294002 and Wortmannin at concentrations of 0.01 μM, 0.1 μM, and 1 μM were stimulated by IL-4 plus anti-CD40 (**a, b**, upper) or LPS (**a, b,** lower). NC, unstimulated, n=3; stimulated without inhibitors, n=7; with DSF, n=3; Bay, n=3; Ibrutinib, n=3; LY294002, n=3; Wortmannin, n=3. Significance was calculated using one-way ANOVA. *$P < 0.05$, **$P < 0.01$, ***$P < 0.001$, ****$P < 0.0001$. compared with the control stimulated without inhibitors, Data are shown as mean ± SEM.

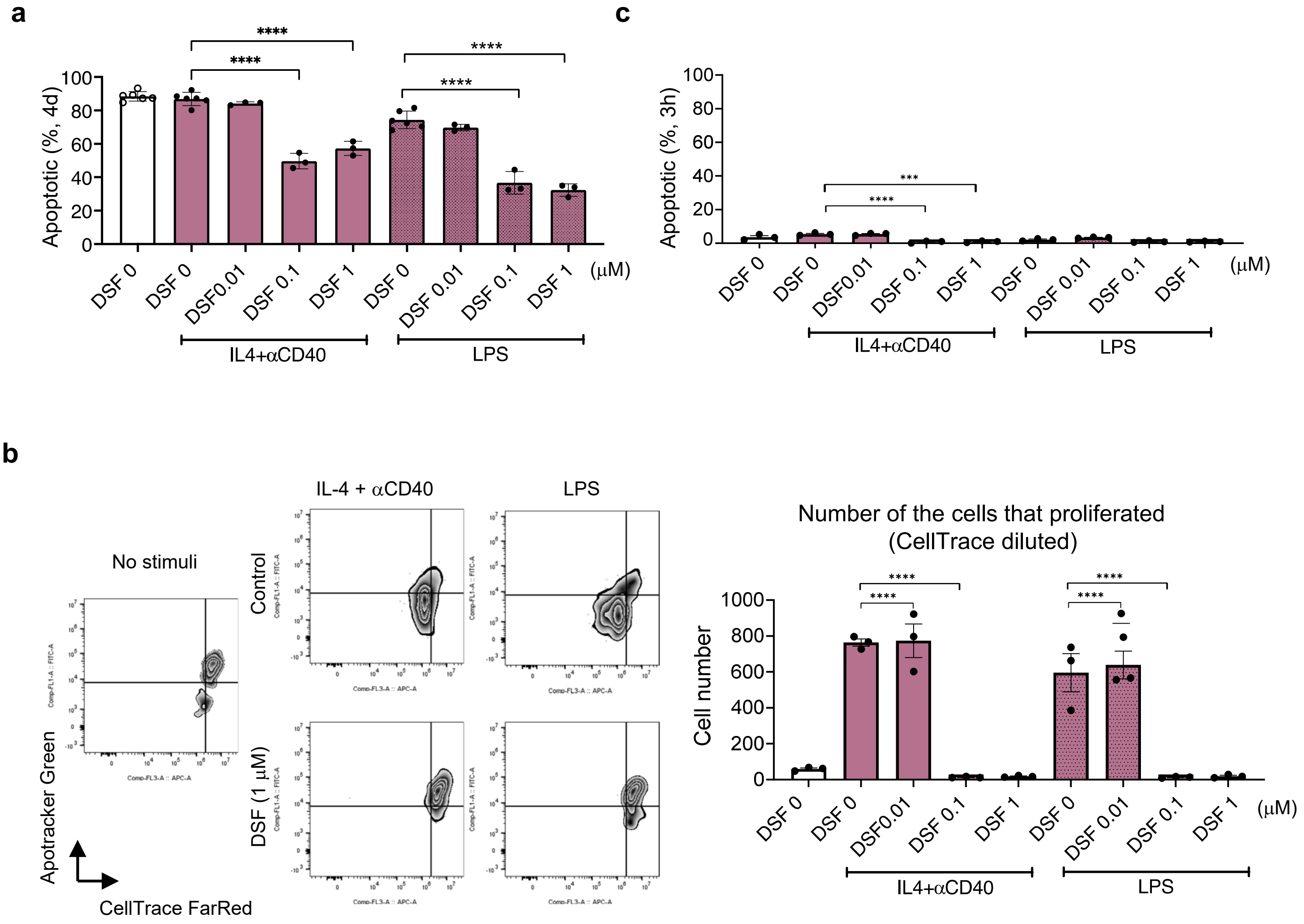

Supplementary Figure 4. The inhibitory effect of DSF on B cells does not depend on inducing B cell apoptosis.
a Percentage of apoptotic cells in B220+ B cells cultured for 4 days and stimulated with IL4+anti-CD40 or LPS in the presence of DSF (NC, unstimulated, n = 6; IL4+anti-CD40, n = 6; LPS, n = 6; DSF, n = 3). **b** Cell proliferation evaluated using CellTrace dilution reagent. Representative flow cytometry plot of CellTrace FarRed versus the apoptosis marker Apotracker (left), and quantitative results of proliferating B cells that exhibited the diluted CellTrace FarRed signal (right) after 48 h of culture.
c Percentage of apoptotic cells in B220+ B cells cultured for 3 h and stimulated with IL4+anti-CD40 or LPS in the presence of DSF (n = 3). *$P < 0.05$, **$P < 0.01$, ***$P < 0.001$, ****$P < 0.0001$ (one-way ANOVA). Data are presented as mean ± SEM.

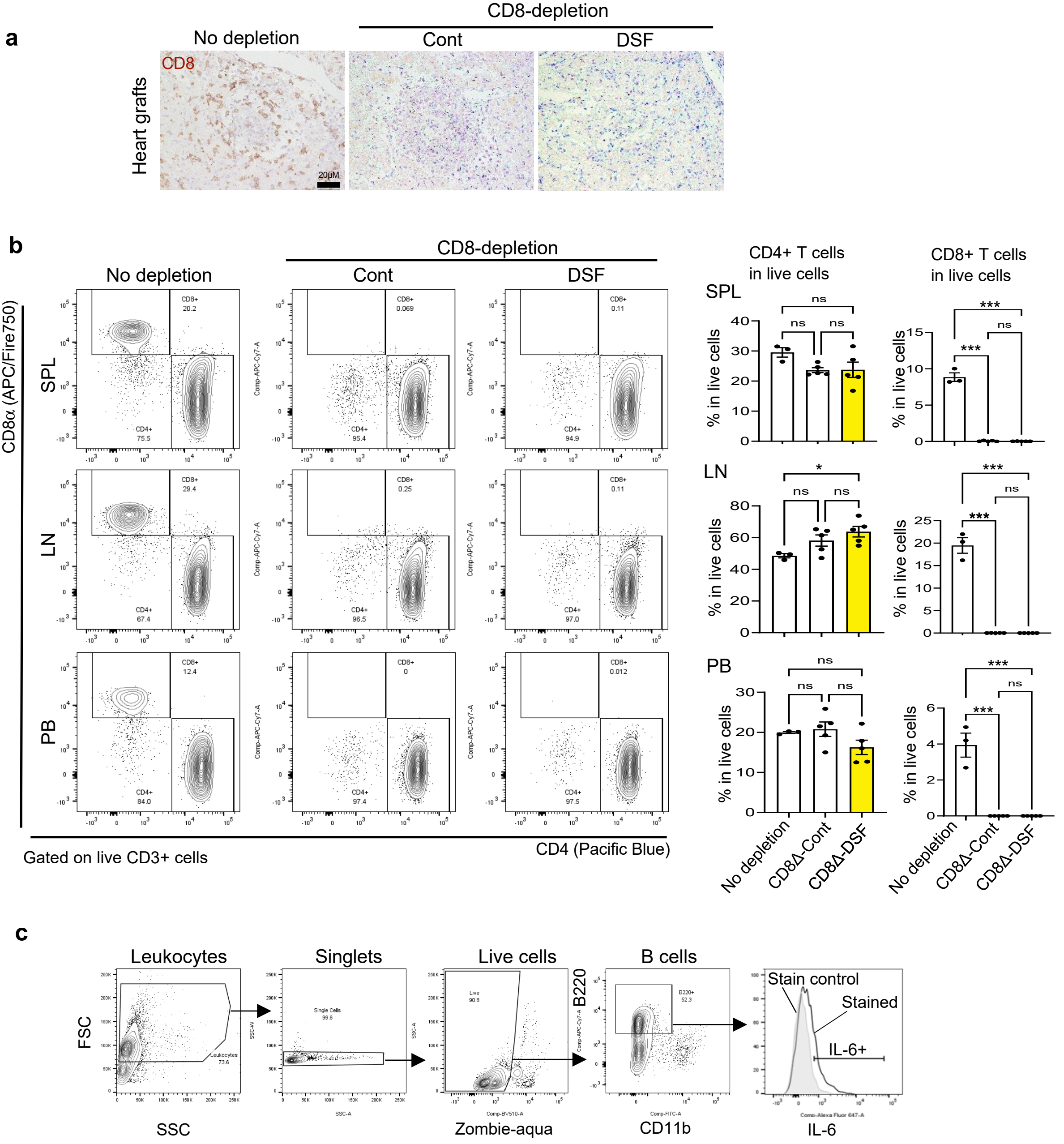
a
No depletion
CD8-depletion
Cont
DSF
Heart grafts
CD8
20μM
b
No depletion
CD8-depletion
Cont
DSF
CD8α (APC/Fire750)
SPL
LN
PB
CD8+
20.2
0.069
0.11
CD4+
75.5
95.4
94.9
29.4
0.25
0.11
67.4
96.5
97.0
12.4
0
0.012
84.0
97.4
97.5
Comp-APC-Cy7-A
Gated on live CD3+ cells
CD4 (Pacific Blue)
CD4+ T cells
in live cells
CD8+ T cells
in live cells
% in live cells
ns
*
***
No depletion
CD8Δ-Cont
CD8Δ-DSF
c
Leukocytes
Singlets
Live cells
B cells
FSC
SSC
Leukocytes
73.6
Single Cells
99.6
SSC-A
SSC-W
Live
90.8
Comp-BV510-A
Zombie-aqua
B220
B220+
52.3
Comp-FITC-A
CD11b
Stain control
Stained
IL-6+
Comp-Alexa Fluor 647-A
IL-6

Supplementary Figure 5. Successful depletion of CD8+ cells using anti-CD8 antibody treatment. a Immunohistochemical analysis of heart grafts, without any depletion or with CD8 depletion using anti-CD8 antibody injection, with staining using anti-CD8 antibody, comparing control diet (Cont) and DSF diet (DSF) groups. **b** Flow cytometry plot of CD4+ or CD8+ T cells gated on live CD3+ cells in the spleen (SPL), lymph nodes (LN), and peripheral blood (PB). Right panels show the percentage of CD4+ or CD8+ T cells in each group. **c** Gating strategy for IL-6+ B220+ cells in the splenocytes isolated from the transplanted recipients on POD9 shown in Fig. 4e. $^{*}P < 0.05$, $^{**}P < 0.01$, $^{***}P < 0.001$; ns, not significant (one-way ANOVA). Data are presented as mean ± SEM.

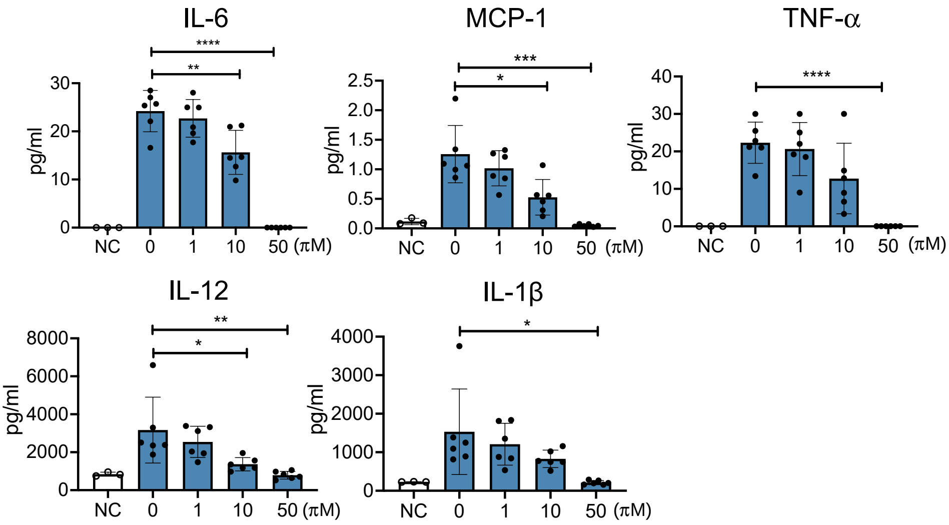

Supplementary Figure 6. Effect of DSF on macrophage cytokine production related to transplant rejection. Effect of DSF on cytokine production by macrophages. The levels of IL-6, MCP-1 (CCL2), TNF-α, IL-12, and IL-1β in macrophage culture supernatants, pretreated with the indicated concentrations of DSF and stimulated with LPS were measured (NC, unstimulated, n=3; LPS, n=6; LPS+DSF, n=6). Significance was calculated using one-way ANOVA: *$P < 0.05$, **$P < 0.01$, ***$P < 0.001$, ****$P < 0.0001$. Data are shown as mean ± SEM.

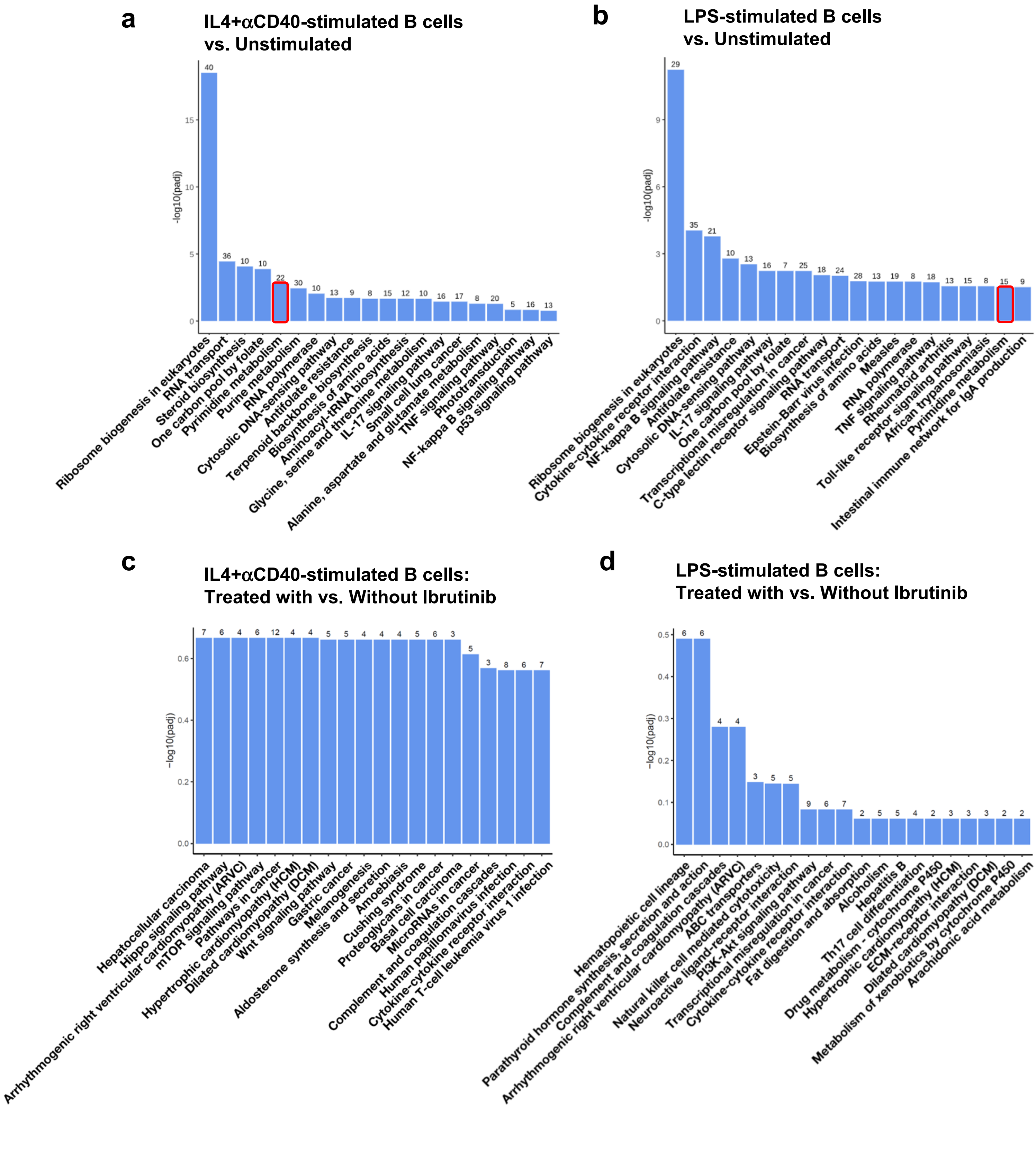

Supplementary Figure 7. Pyrimidine metabolism pathway was moderately activated in the B cells under stimulation, but no significant inhibitory effect was shown under Ibrutinib treatment. a,b KEGG pathway (up-regulated genes) enrichment analysis of RNA-seq data of B cells 3 h after stimulation with IL4+anti-CD40 antibody (**a**) or LPS (**b**) compared with unstimulated B cells , **c,d** pathways downregulated in Ibrutinib-pre-treated B cells compared with those in B cells without Ibrutinib 3 h after stimulation with IL4+anti-CD40 antibody (**c**) or LPS (**d**).
