# Supplementary material for: Disulfiram treatment suppresses antibody-producing reactions by inhibiting macrophage activation and B cell pyrimidine metabolism
Source: Commun Biol. 2024 Apr 22;7:488. doi: 10.1038/s42003-024-06183-9 (PMC11035657; doi:10.1038/s42003-024-06183-9)
Supplement: Supplementary file 4 — Reporting summary [file 42003_2024_6183_MOESM4_ESM.pdf]

nature portfolio

Corresponding author(s): Etsuko Toda; Akira Shimizu

Last updated by author(s): Apr 5, 2024

Reporting Summary

Nature Portfolio wishes to improve the reproducibility of the work that we publish. This form provides structure for consistency and transparency in reporting. For further information on Nature Portfolio policies, see our Editorial Policies and the Editorial Policy Checklist.

Statistics

For all statistical analyses, confirm that the following items are present in the figure legend, table legend, main text, or Methods section.

n/a	Confirmed	
☐	☒	The exact sample size (n) for each experimental group/condition, given as a discrete number and unit of measurement
☐	☒	A statement on whether measurements were taken from distinct samples or whether the same sample was measured repeatedly
☐	☒	The statistical test(s) used AND whether they are one- or two-sided<br>*Only common tests should be described solely by name; describe more complex techniques in the Methods section.*
☒	☐	A description of all covariates tested
☒	☐	A description of any assumptions or corrections, such as tests of normality and adjustment for multiple comparisons
☐	☒	A full description of the statistical parameters including central tendency (e.g. means) or other basic estimates (e.g. regression coefficient) AND variation (e.g. standard deviation) or associated estimates of uncertainty (e.g. confidence intervals)
☐	☒	For null hypothesis testing, the test statistic (e.g. F, t, r) with confidence intervals, effect sizes, degrees of freedom and P value noted<br>*Give P values as exact values whenever suitable.*
☒	☐	For Bayesian analysis, information on the choice of priors and Markov chain Monte Carlo settings
☒	☐	For hierarchical and complex designs, identification of the appropriate level for tests and full reporting of outcomes
☒	☐	Estimates of effect sizes (e.g. Cohen's d, Pearson's r), indicating how they were calculated

Our web collection on statistics for biologists contains articles on many of the points above.

Software and code

Policy information about availability of computer code

Data collection	cellSense software (Olympus), CytExpert software (Beckman Coulter), BD FACSDiva software (BD Biosciences)
Data analysis	FlowJo software v10.6.2(BD Biosciences), GraphPad Prism version 9 (GraphPad Software Inc.),<br>QuPath software (version 0.3.2), WinROOF software (2015), ClustVis 51 (https://biit.cs.ut.ee/clustvis/)

For manuscripts utilizing custom algorithms or software that are central to the research but not yet described in published literature, software must be made available to editors and reviewers. We strongly encourage code deposition in a community repository (e.g. GitHub). See the Nature Portfolio guidelines for submitting code & software for further information.

Data

Policy information about availability of data

All manuscripts must include a data availability statement. This statement should provide the following information, where applicable:

- Accession codes, unique identifiers, or web links for publicly available datasets
- A description of any restrictions on data availability
- For clinical datasets or third party data, please ensure that the statement adheres to our policy

RNA sequencing data have been deposited in the NCBI GEO and are accessible through the accession number GSE261712.
The source data underlying the graphs in the article and supplementary information file can be found in Supplementary Data.
Any further detail is available from the corresponding author on reasonable request.

Research involving human participants, their data, or biological material

Policy information about studies with human participants or human data. See also policy information about sex, gender (identity/presentation), and sexual orientation and race, ethnicity and racism.

Reporting on sex and gender	n/a
Reporting on race, ethnicity, or other socially relevant groupings	n/a
Population characteristics	n/a
Recruitment	n/a
Ethics oversight	n/a

Note that full information on the approval of the study protocol must also be provided in the manuscript.

Field-specific reporting

Please select the one below that is the best fit for your research. If you are not sure, read the appropriate sections before making your selection.

☒ Life sciences ☐ Behavioural & social sciences ☐ Ecological, evolutionary & environmental sciences

For a reference copy of the document with all sections, see nature.com/documents/nr-reporting-summary-flat.pdf

Life sciences study design

All studies must disclose on these points even when the disclosure is negative.

Sample size	Sample size was determined based on the numbers reported in the field.
Data exclusions	No data were excluded.
Replication	All experiments were repeated multiple times.
Randomization	Mice were randomly grouped into control and treatment groups.
Blinding	Investigators were not blinded to mouse treatments during experiments.

Reporting for specific materials, systems and methods

We require information from authors about some types of materials, experimental systems and methods used in many studies. Here, indicate whether each material, system or method listed is relevant to your study. If you are not sure if a list item applies to your research, read the appropriate section before selecting a response.

Materials & experimental systems

n/a	Involved in the study	
☐	☒	Antibodies
☒	☐	Eukaryotic cell lines
☒	☐	Palaeontology and archaeology
☐	☒	Animals and other organisms
☒	☐	Clinical data
☒	☐	Dual use research of concern
☒	☐	Plants

Methods

n/a	Involved in the study	
☒	☐	ChIP-seq
☐	☒	Flow cytometry
☒	☐	MRI-based neuroimaging

Antibodies

Antibodies used	The antibodies used in these studies were anti-CD8 mAb (BE0061, clone 2.43, BioXCell, NH, USA) for CD8-positive cell depletion, C4d (HP1088, dilution 1:100, Hycult Biotech, USA) for detecting complement, F4/80 (70076, clone D2S9R, dilution 1:100, Cell Signaling Technology, Beverly, MA, USA) for detecting graft-migrated macrophages, and CD8 (ab209775, clone EPR20305, dilution 1:100, Abcam, Cambridge, UK) , Ki67 (718071, clone sp6, ready-to-use, Nichirei Biosciences, Japan), B220 (103202, clone RA3-6B2, dilution

1:100, BioLegend, Japan), Alexa Fluor 594 conjugated CD31 (102432, clone 390, dilution 1:100, BioLegend), Alexa Fluor 480-conjugated IgM (ab150121, dilution 1:200, Abcam), and Alexa Fluor 480-conjugated IgG (a-21202, dilution 1:200, Thermo Fisher Scientific, Waltham, MA, USA),
anti-mouse CD4 antibody (ab237722, clone CAL4, dilution 1:100, Abcam) and Foxp3 (14-5773-82, clone FJK-16s, dilution 1:100, eBioscience, San Diego, CA, USA), followed by staining with Alexa Fluor 488-conjugated anti-rabbit IgG antibody (A21202, dilution 1:200, Thermo Fisher Scientific) and Alexa Fluor 594-conjugated anti-rat IgG (A21209, dilution 1:200, Thermo Fisher Scientific) for immunofluorescent staining,
anti-CD16/CD32 antibody (BE0307, clone 2.4G2, Bio X Cell) for blocking Fc receptor at 1:100,
anti-mouse antibodies: CD3ε -PE/Dazzle 594 (clone 145-2C11, dilution 1:200, Cat 100347), CD4-Pacific blue (clone RM4-4, dilution 1:200, Cat 116008), B220-AlexaFluor700 (clone RA3-6B2, dilution 1:200, Cat 103232), CD138-PE (clone 281-2, dilution 1:200 Cat 142504), and CD8- APC 750 (clone 53-6.7, dilution 1:200 Cat 100766).
IL-6-APC (clone RM4-4, dilution 1:200, BioLegend, Cat 116008), anti-Foxp3-Alexa Fluor 488 and isotype control antibodies (eBioscience), CD4-Pacific blue (clone RM4-4, dilution 1:200, Cat 116008), CD25-PE (clone 7D4, dilution 1:200, BD biosciences, Cat 558642), CD3ε -PE/Dazzle 594 (clone 145-2C11, dilution 1:100, Cat 100347) and CD45-APC (clone 30-F11, dilution 1:400, BioLegend, Cat 103112), Alexa Fluor 488-conjugated anti-IgG (A-21202, Thermo Fisher, 1:150) or Alexa Fluor 488-conjugated anti-IgM (ab150121, Abcam, 1:150) for flow cytometry, and anti-CD40 (clone 1C10; BioLegend) for stimulating B cells.

Validation: The antibodies have been validated by the vendors or by our previous published work.

Animals and other research organisms

Policy information about studies involving animals; ARRIVE guidelines recommended for reporting animal research, and Sex and Gender in Research

Laboratory animals	Description of research mice used for experiments can be found in the relevant figure legends and Methods.
Wild animals	Not used.
Reporting on sex	Description of sex can be found in the relevant Methods.
Field-collected samples	Not used.
Ethics oversight	All animal experiments were approved by the Animal Experiments Ethical Review Committee of Nippon Medical School (Tokyo, Japan, No. 2019-048)

Note that full information on the approval of the study protocol must also be provided in the manuscript.

Flow Cytometry

Plots

Confirm that:

- [x] The axis labels state the marker and fluorochrome used (e.g. CD4-FITC).
- [x] The axis scales are clearly visible. Include numbers along axes only for bottom left plot of group (a 'group' is an analysis of identical markers).
- [x] All plots are contour plots with outliers or pseudocolor plots.
- [x] A numerical value for number of cells or percentage (with statistics) is provided.

Methodology

Sample preparation	See methods
Instrument	Cytoflex; Beckman Coulter Inc., LSRFortessa; BD Biosciences
Software	FlowJo software 10.6.2 (BD Biosciences)
Cell population abundance	See methods
Gating strategy	See methods

- [x] Tick this box to confirm that a figure exemplifying the gating strategy is provided in the Supplementary Information.
